# Human papilloma virus (HPV) integration signature in Cervical Cancer: identification of *MACROD2* gene as HPV hot spot integration site

**DOI:** 10.1038/s41416-020-01153-4

**Published:** 2020-11-16

**Authors:** Maud Kamal, Sonia Lameiras, Marc Deloger, Adeline Morel, Sophie Vacher, Charlotte Lecerf, Célia Dupain, Emmanuelle Jeannot, Elodie Girard, Sylvain Baulande, Coraline Dubot, Gemma Kenter, Ekaterina S. Jordanova, Els M. J. J. Berns, Guillaume Bataillon, Marina Popovic, Roman Rouzier, Wulfran Cacheux, Christophe Le Tourneau, Alain Nicolas, Nicolas Servant, Suzy M. Scholl, Ivan Bièche, RAIDs Consortium, Anne de la Rochefordiere, Pierre Fumoleau, Aljosa Mandic, Nina Samet, Choumouss Kamoun, Windy Rondoff, Sebastien Armanet, Alexandra Rohel, Souhir Neffati, Marie-Emmanuelle Legrier, Sinette Ngoumou Mabiala, Sylvain Dureau, Coralie Errera, Marius Craina, Madalin Margan, Sanne Samuels, Henry Zijlmans, Peter Hillemanns, Sorin Dema, Alis Dema, Goran Malenkovic, Branislav Djuran, Anne Floquet, Frédéric Guyon, Pierre Emmanuel Colombo, Michel Fabbro, Christine Kerr, Charlotte Ngo, Fabrice Lecuru, Eleonor Rivin del Campo, Charles Coutant, Frédéric Marchal, Nathalie Mesgouez-Nebout, Virginie Fourchotte, Jean Guillaume Feron, Philippe Morice, Eric Deutsch, Pauline Wimberger, Jean-Marc Classe, Heiko von der Leyen, Mathieu Minsat, Istvan Nagy, Balazs Balint, Nicolas de Saint-Jorre, Alexia Savignoni, Franck Perez, Patricia Tresca, Noreen Gleeson, Philippe Hupe, Sergio Roman Roman, Emmanuel Barillot, Fanny Coffin, Bastiaan Nuijen, Alexandre Boissonnas, Marc Billaud, Laurence Lafanechere, Jaap Verweij, Arjan Bandel, Jozien Hellemann, Kirsten Ruigrok-Ritstier, Philipp Harter, Christian Kurzeder, Alexander Mustea, Eugeniu Banu, Elisabeta Patcas, Victor Cernat, Andrea Slocker, Michele Mondini, Maud Bossard, Julie Chupin, Sjoerd Rodenhuis, Rene Medema, Anika Havemeier, Thomas Fink, Amelie Michon, Christine Kubiak, Corine Beaufort, Judit Cseklye, Dora Latinovics, Peter Bihari, Isabel Brito, Bérengère Ouine, Leanne De Koning, Vincent Puard, Elaine Del Nery, Jos Beijnen, Dominique Koensgen, Daniela Bruennert, Milos Lucic, Natalja ter Haar

**Affiliations:** 1 Department of Drug Development and Innovation, Institut Curie, PSL Research University, 75005 Paris & 92210, Saint-Cloud, France; 2 Department of Drug Development and Innovation, Institut Curie, PSL Research University, 92210 Saint-Cloud, France; 3 Institut Curie, Genomics of Excellence (ICGex) Platform, PSL Research University, 75005 Paris, France; 4 Bioinformatics and Computational Systems Biology of Cancer, PSL Research University, Mines Paris Tech, INSERM U900, 75005 Paris, France; 5 Department of Genetics, Institut Curie, PSL Research University, 75005 Paris, France; 6 Department of Pathology, Institut Curie, PSL Research University, 75005 Paris, France; 7 Center for Gynaecologic Oncology Amsterdam, Amsterdam UMC and The Netherlands Cancer Institute - Antoni van Leeuwenhoek Hospital, Amsterdam, The Netherlands; 8 Department of Pathology, Leiden University Medical Center, Leiden, The Netherlands; 9 Department of Medical Oncology, Erasmus MC, 3000 CA Rotterdam, The Netherlands; 10 Oncology Institute of Vojvodina, Put doktora Goldmana, 421204 Sremska Kamenica, Serbia; 11 Department of Surgery, Institut Curie, PSL Research University, 92210 Saint-Cloud, France; 12 Paris-Saclay University, Paris, France; 13 Hopital Privé Pays de Savoie, Service d’oncologie médicale, 19 avenue Pierre Mendès France, 74100 Annemasse, France; 14 Institut Curie, PSL Research University, CNRS UMR3244, 75248 Paris, France; 15 Faculty of Pharmaceutical and Biological Sciences, INSERM U1016, Paris Descartes University, 75005 Paris, France; 16 Department of Drug Development and Innovation, Institut Curie, PSL Research University, Paris, France; 17 Institut Curie, Paris, France; 18 Gynecologic Oncology Department Clinic for Operative Oncology, Institute of Oncology of Vojvodina, Sremska, Serbia; 19 Publica Institutul Oncologic, Chișinău, Republic of Moldova; 20 Gustave Roussy, Paris, France; 21 Arcagy-Gineco, Paris, France; 22 Insitut Pasteur, Paris, France; 23 Quanticsoft, Paris, France; 24 University of Medicine and Pharmacy “Victor Babeș”, Timișoara, Romania; 25 Center for Gynaecologic Oncology Amsterdam, Amsterdam UMC and The Netherlands Cancer Institute - Antoni van Leeuwenhoek Hospital, Amsterdam, Netherlands; 26 Hannover Medical School, Hanover, Germany; 27 Chirurgie onco-gynécologique and Oncology, Institut Bergonié, Centre Régional de Lutte contre le Cancer Bordeaux-Aquitaine, Bordeaux, France; 28 Centre Val d’Aurelle, Paris, France; 29 Service de chirurgie cancérologique gynécologique et du sein, Hôpital Européen Georges Pompidou, APHP et faculté de médecine, Université Paris Descartes, Paris, France; 30 Department of Surgery, Institut Curie, PSL Research University, 75005 Paris, France; 31 Department of Surgery, Institut Curie, PSL Research University, 92210 Saint-Cloud, France; 32 Department of Radiation Oncology, Tenon University Hospital, Hôpitaux Universitaires Est Parisien, Sorbonne University Medical Faculty, Paris, France; 33 Centre Georges François Leclerc, Paris, France; 34 Département de chirurgie, CRAN, UMR 7039, Université de Lorraine, CNRS, Institut de Cancérologie de Lorraine, Vandœuvre-lès-Nancy, France; 35 Institut de cancérologie de l’Ouest - site Paul Papin (ICO), Paris, France; 36 Department of Gynecology and Obstetrics, Universitätsklinikum Carl Gustav Carus; an der Technischen Universität Dresden, Fetscherstraße 74, 01307 Dresden, Germany; 37 René Gauducheau, Paris, France; 38 Hannover Clinical Trial Center GmbH, Hannover, Germany; 39 SeqOmics Biotechnology Ltd, Vallalkozok utja 7, Morahalom, Hungary; 40 Institut Curie, PSL Research University, Paris, France; 41 Insitut Curie, Paris, France; 42 St James/Trinity College, Dublin, Ireland; 43 Netherlands Cancer Institute, Amsterdam, The Netherlands; 44 Université Pierre et Marie Curie, Paris, France; 45 Université Joseph-Fourier, Grenoble, France; 46 Erasmus University Medical Centre, Rotterdam, Netherlands; 47 Department of Medical Oncology, Erasmus MC Cancer Institute, Erasmus University Medical Center, 3015 CN Rotterdam, Netherlands; 48 Department of Gynecology and Gynecologic Oncology, Ev. Kliniken Essen-Mitte, Essen, Germany; 49 Department of Obstetrics and Gynecology, University Hospital of Basel, Basel, Switzerland; 50 Department of Gynecology and Gynecological Oncology, University Hospital, 53127 Bonn, Germany; 51 Spitalul Sfantul Constantin Brasov, Brasov, Romania; 52 Department of Gynecology and Obstetrics, University Medicine Greifswald, Greifswald, Germany; 53 Ayming, Gennevilliers, France; 54 European Clinical Research Infrastructure Network, Toulouse, France; 55 University of Greifswald, Greifswald, Germany; 56 Oncology Institute of Vojvodina, Diagnostic Imaging Centre, University of Novi Sad, University School of Medicine, Put Dr. Goldmana 4, Sremska Kamenica, 21204 Novi Sad, Serbia

**Keywords:** Oncology, Molecular medicine, Biomarkers, Molecular biology

## Abstract

**Background:**

Cervical cancer (CC) remains a leading cause of gynaecological cancer-related mortality with infection by human papilloma virus (HPV) being the most important risk factor. We analysed the association between different viral integration signatures, clinical parameters and outcome in pre-treated CCs.

**Methods:**

Different integration signatures were identified using HPV double capture followed by next-generation sequencing (NGS) in 272 CC patients from the BioRAIDs study [NCT02428842]. Correlations between HPV integration signatures and clinical, biological and molecular features were assessed.

**Results:**

Episomal HPV was much less frequent in CC as compared to anal carcinoma (*p* < 0.0001). We identified >300 different HPV-chromosomal junctions (inter- or intra-genic). The most frequent integration site in CC was in *MACROD2* gene followed by *MIPOL1/TTC6* and *TP63*. HPV integration signatures were not associated with histological subtype, FIGO staging, treatment or PFS. HPVs were more frequently episomal in *PIK3CA* mutated tumours (*p* = 0.023). Viral integration type was dependent on HPV genotype (*p* < 0.0001); HPV18 and HPV45 being always integrated. High HPV copy number was associated with longer PFS (*p* = 0.011).

**Conclusions:**

This is to our knowledge the first study assessing the prognostic value of HPV integration in a prospectively annotated CC cohort, which detects a hotspot of HPV integration at *MACROD2*; involved in impaired PARP1 activity and chromosome instability.

## Background

Cervical cancer (CC) remains a leading cause of gynaecological cancer-related mortality worldwide and constitutes the second most common malignancy in women.^1^ Although patients with CC exhibit differences in clinical behaviour, infection by high-risk human papilloma virus (HPV) remains an important initiating event in CC tumorigenesis,^2^ and one of the most important risk factors for developing CC.^3^ Most HPV infections are cleared spontaneously by the immune system, yet in some cases, it persists leading to cancer.^4^ Following infection, the virus can remain in its episomal form, or become integrated into the host genome. Both patterns may be present jointly (episomal/integrated).^5^ It is thought that the longer half-life of integrated viral transcripts compared to half-life of episomal transcripts favours cellular immortalisation and transformation into cancer cells while also providing a selective growth advantage.^6^ Most often, the integration of HPV DNA leads to a breakpoint in the E2 gene, resulting in de-repression of the E6 and E7 viral oncogenes. When the virus remains episomal, expression of E6 and E7 proteins may result from leaky expression or epigenetics dysregulation. E6 and E7 proteins impact the function of p53 and pRb proteins, allowing squamous cell tumorigenesis.^6^

Several mechanisms of integration have been reported in the literature; the “looping” model of HPV integration following DNA replication and recombination (resulting in DNA concatemers)^7^ is the most widely accepted but not experimentally reconstituted. HPV DNA integration into the human genome triggers various genetic alterations, such as oncogenes amplification, tumour suppressor gene inactivation, inter- or intra- chromosomal rearrangements as well as genetic instability.^6,8^ Genes localised near the integration sites of viral genomes can experience changes in RNA and protein expression levels, leading to over- or under-expression. In 2015, whole-genome sequencing and high-throughput viral integration methods identified as many as 3667 HPV integration breakpoints in cervical neoplastic lesions. Frequent integration sites have been reported in genes relevant to the neoplastic process, such as the *MYC* oncogene.^9^ Loss of function (LOF) in the *RAD51B* tumour suppressor gene following HPV DNA insertion was reported to affect the DNA repair pathway and genomic instability in CC.^10^

HPV DNA integration occurs as a single copy or in multiple repeats (in tandem or dispersed).^11^ In 2016, Holmes et al. developed a Capture HPV method to identify five different HPV signatures in 72 CC. The first two signatures contain two hybrid chromosomal–HPV junctions which are co-linear (2 Junctions Colinear “2J-COL”) or non-linear (2 Junctions Non-Linear “2J-NL”) depending on their relative orientations. It reflects two modes of viral integration, associated with chromosomal deletion or amplification events, respectively. The third and fourth signatures exhibit several hybrid junctions either clustered in one chromosomal region (Multiple Junctions Clustered “MJ-CL”) or scattered at distinct loci (Multiple Junctions Scattered “MJ-SC”) while the fifth signature consists of episomal forms of HPV (EPI).^12^

On the assumption that HPV integration types/signatures/pattern might predict clinical outcomes, we analysed the association between the different viral integration signatures, clinical and pathological parameters and outcome in the large cohort of 272 HPV-positive CC patients enrolled in the prospective BioRAIDs study [NCT02428842].

## Methods

### Patients and samples

Patients included in this study were enrolled in the EU-funded RAIDs Network (Rational Molecular Assessment and Innovative Drug Selection, www.raids-fp7.eu) prospective CC BioRAIDs study [NCT02428842]. The clinical protocol together with tumour sampling procedures, quality control of samples and treatment in 18 European centres (seven European countries) as well as study results have been previously published.^13–15^

### HPV typing

All samples included in this study were analysed for HPV type, using the SPF10 primer set and INNO-LiPA HPV genotyping extra line probe assay (Fujirebio Europe, Gent, Belgium) according to the manufacturers’ protocol. For DNA isolation, one to five 10 μm tissue sections were cut depending on the size of the tumour biopsy. DNA was isolated using the automated Tissue Preparation System (Siemens Healthcare Diagnostics, NY, USA).

### *PIK3CA* mutation detection

A mutational analysis of the *PIK3CA* gene had been previously carried out on all tumour samples.^15^ In summary, paired-end whole-exome sequencing was performed on a HiSeq2500 platform, with an Agilent SureSelectXT Human. The sequencing was performed to reach an average depth of coverage of at least 80× per sample. Dedicated filtering strategies were applied to somatic variants depending on their functional impact per gene category: oncogene or tumour suppressor gene or uncharacterised. For oncogenes as *PIK3CA*, hotspot missense mutations known in the COSMIC database were considered. Among the 87 *PIK3CA* mutations, three patients had an H1047R mutation (exon 20) and 84 patients had a E452K/E545K mutation (exon 9).

### DNA library preparation

The DNA libraries were prepared using 500 ng of genomic DNA (extracted from frozen tissue), starting with ultra-sonication (Covaris) to produce double-strand DNA fragments of approximately 280 bp. End-Repair and A-tailing were applied to facilitate ligation of the adapters, containing unique barcodes for each sample, specific to the Illumina technology for amplification and sequencing. KAPA Hyper Prep kit was used, according to the manufacturer’s instructions.

### HPV double capture method

The double capture method was carried out using the SeqCap EZ Rapid Library Small Target Capture method, developed by Roche, which is adapted to capture small DNA targets. The DNA libraries were multiplexed (by 12) and hybridised for 16 h with the biotinylated HPV oligonucleotide probes, recognising all HPV genotypes. The DNA sequences were then captured by streptavidin beads and amplified by PCR. We performed a double capture (i.e. two rounds of hybridisation and capture) to improve the efficiency and specificity. Post-capture libraries were sequenced using Illumina MiSeq system (Illumina, San Diego, CA, USA), in paired-end 150, with 24 samples multiplexed on a V2 micro flow-cell.

The HPV copy number shows the abundance of the target relative to the endogenous control (*KLK3*) in order to normalise the starting amount and quality of genomic DNA. Similar results were obtained with other endogenous diploid controls (*GAPDH*, *RAB7A*).

### Bioinformatics analyses

In order to analyse our HPV capture data, we set up a new bioinformatics pipeline called nf-VIF available at https://github.com/bioinfo-pf-curie/nf-vif/, which implements the methods we already described in Holmes et al. Briefly nf-VIF performs (i) quality controls and cleaning of raw sequencing Illumina data, (ii) HPV genotyping, and (iii) the detection of the HPV insertion sites within the human genome. Nf-VIF is implemented through the Nextflow workflow management system, ensuring a high portability, reproducibility, and scalability (see Supplementary materials for details).

### Statistical analysis

The correlations between HPV integration signatures and clinical, biological and molecular features were analysed using chi-square tests, chi-square tests with Yates’ correction or Fisher’s exact tests, as appropriate. Progression-free survival (PFS) was defined as the time interval from the date of CC diagnosis to progression. Survival data were censored on the date of last follow-up. To visualise the efficacy of a molecular marker (i.e., HPV copy number) to discriminate two populations (patients who progressed) in the absence of an arbitrary cut-off value, data were summarised in an ROC curve.^16,17^ The AUC (area under curve) was calculated as a single measure to discriminate efficacy. Survival curves were estimated by the Kaplan−Meier method, and compared using the log-rank test. For all statistical tests, significance level was defined as *p* < 0.05.

## Results

### Patient characteristics

Clinical, histological, biological (including *PIK3CA* mutational status) and outcome of the 272 HPV-positive CC patients from the BioRAIDs European study are presented in Table 1. All samples were obtained prior to treatment. Median PFS of the whole cohort was 20.15 months. Fifty-four (20%) patients were treated with upfront surgery, 42 (15%) patients with neoadjuvant chemotherapy and 176 (65%) patients with external beam radiation therapy with concomitant platinum-based chemotherapy. The majority of patients (230 patients corresponding to 85%) had squamous cell carcinoma. Classical prognostic biomarkers such as FIGO stage (2018) and presence of lymph nodes (FIGO III/IV) correlated with PFS in the study population (Table 1).

**Table 1:** Clinical and biological characteristics of 272 patients with HPV-positive cervical cancer, in relation to progression-free survival.

	Patients (%)	Events (%)^a^	PFS (*p* value)^b^
Total	272 (100.0)	84 (30.9)	
Age			
≤50	140 (51.5)	41 (29.3)	0.40 (NS)
>50	132 (48.5)	43 (32.6)	
Histologic subtype			
Squamous cell carcinoma	230 (84.6)	71 (30.9)	0.56 (NS)
Adenocarcinoma	27 (9.9)	8 (29.6)	
Adenosquamous carcinoma	10 (3.7)	4 (40.0)	
Mixed form or undifferentiated	5 (1.8)	1 (20.0)	
HPV status			
Genotype 16	155 (57.0)	50 (32.3)	0.13 (NS)
Genotype 18	36 (13.2)	14 (38.9)	
Genotype 45	27 (9.9)	10 (37.0)	
Genotype 31	9 (3.3)	0 (0)	
Genotype 33	11 (4.0)	0 (0)	
Other genotypes^c^	34 (12.5)	10 (29.4)	
FIGO stage			
I/II	205 (75.4)	50 (24.4)	<**0.0001**
III/IV	67 (24.6)	34 (50.7)	
Nodal involvement			
Yes	167 (61.4)	62 (37.1)	**0.0028**
No	105 (38.6)	22 (21.0)	
Pelvic lymph nodes			
Yes	165 (60.7)	62 (37.6)	**0.0015**
No	107 (39.3)	22 (20.6)	
Para-aortic lymph nodes			
Yes	43 (15.8)	22 (51.2)	**0.0001**
No	229 (84.2)	62 (27.1)	
Initial therapy			
Surgery	54 (19.9)	10 (18.5)	**0.0008**
Radiotherapy	176 (64.7)	52 (29.5)	
Neoadjuvant chemotherapy	42 (15.4)	22 (52.4)	
PIK3CA mutational status^d^			
WT	182 (67.7)	60 (33.0)	0.23 (NS)
Mutated	87 (32.3)	23 (26.4)	

Significant results are displayed in bold.

*NS* not significant.

^a^Until 24 months.

^b^Log-rank test.

^c^Other HPV genotypes: 39, 42, 52, 56, 58, 59, 68, 70, 73, 82.

^d^Information available for 269 patients.

HPV16 was the most common genotype (*n* = 155, 57%) followed by HPV18 (*n* = 36, 13%) and HPV45 (*n* = 27, 10%) (Table 1). Eighty-seven patients (32%) harboured a *PIK3CA* mutation, which on its own did not correlate to PFS in this subpopulation.

### Integration mechanisms

**Fig. 1:**
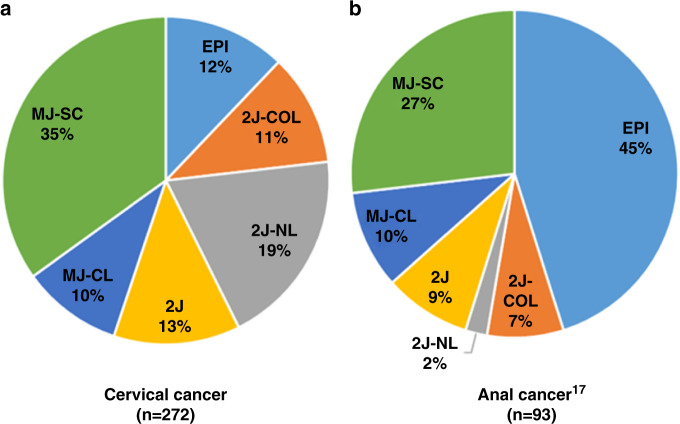
Distribution of the HPV integration signatures according to the location of HPV-positive squamous cell carcinomas. **a** Cervical cancer; **b** anal cancer; *p* < 0.0001. 2J-COL 2 Junctions Colinear, 2J-NL 2 Junctions Non-Linear, MJ-CL Multiple Junctions Clustered, MJ-SC Multiple Junctions Scattered, EPI episomal and 2J 2 Junctions.

The breakpoints identified on the HPV genome and HPV statuses are reported in Table 2 and Supplementary Table 1. In the absence of integration (*n* = 33, 12%), no HPV-chromosomal breakpoint was observed and the viral genome persisted in an episomal form (EPI). Five HPV integration patterns were observed: 2J-COL (*n* = 30, 11%), 2J-NL (*n* = 53, 20%), 2J (*n* = 34, 12%), MJ-CL (*n* = 27, 10%), MJ-SC (*n* = 95, 35%). The BioRAIDs CC series differed significantly from that of HPV-positive anal carcinoma (*p* < 0.0001) (Fig. 1) recently published by our team^17^ in that episomal HPV was much less frequent in CC as compared to anal carcinoma, while “2J” signatures (2J and 2J-NL and 2J-CPL) were more often represented in CC. The results were similar in HPV16-positive cancers that represent the majority of the subtypes in both cervical and anal cancers (Supplementary Fig. 1).

**Table 2:** Relationship between mechanisms of integration of HPV and clinical, biological and pathological characteristics of the 272 patients with HPV-positive cervical cancer.

		Number of patients (%)			
HPV insertion^a^	Patients (%)	EPI	2J	MJ	*p* value^b^
Total	272 (100.0)	33 (12.1)	117 (43.0)	122 (44.9)	
Age					
≤50	140 (51.5)	11 (33.3)	60 (51.3)	69 (56.6)	0.061 (NS)
>50	132 (48.5)	22 (66.7)	57 (48.7)	53 (43.4)	
Histologic subtype					
Squamous cell carcinoma	230 (84.6)	27 (81.8)	92 (78.6)	111 (91.1)	0.14 (NS)
Adenocarcinoma	27 (9.9)	3 (9.1)	18 (15.4)	6 (4.9)	
Adenosquamous carcinoma	10 (3.7)	2 (6.1)	4 (3.4)	4 (3.3)	
Mixed form or undifferenciated	5 (1.8)	1 (3.0)	3 (2.6)	1 (0.8)	
HPV status					
Genotype 16	155 (57.0)	25 (75.8)	44 (37.6)	86 (70.5)	**<0.0001**
Genotype 18	36 (13.2)	0 (0.0)	24 (20.5)	12 (9.8)	
Genotype 45	27 (9.9)	0 (0.0)	24 (20.5)	3 (2.5)	
Genotype 31	9 (3.3)	2 (6.1)	4 (3.4)	3 (2.5)	
Genotype 33	11 (4.0)	3 (9.1)	4 (3.4)	4 (3.3)	
Other genotypes^c^	34 (12.5)	3 (9.1)	17 (14.5)	14 (11.5)	
Stage FIGO					
I/II	205 (75.4)	25 (75.8)	86 (73.5)	94 (77.0)	0.82 (NS)
III/IV	67 (24.6)	8 (24.2)	31 (26.5)	28 (23.0)	
Lymph node					
Yes	167 (61.4)	19 (57.6)	81 (69.2)	67 (54.9)	0.067 (NS)
No	105 (38.6)	14 (42.4)	36 (30.8)	55 (45.1)	
Pelvis lymph node					
Yes	165 (60.7)	19 (57.6)	80 (68.4)	66 (54.1)	0.072 (NS)
No	107 (39.3)	14 (42.4)	37 (31.6)	56 (45.9)	
Para-aortic lymph node					
Yes	43 (15.8)	3 (9.1)	20 (17.1)	20 (16.4)	0.52 (NS)
No	229 (84.2)	30 (90.9)	97 (82.9)	102 (83.6)	
Initial therapy					
Surgery	54 (19.9)	7 (21.2)	21 (17.9)	26 (21.3)	0.86 (NS)
Radiotherapy	176 (64.7)	22 (66.7)	75 (64.1)	79 (64.8)	
Neoadjuvant chemotherapy	42 (15.4)	4 (12.1)	21 (17.9)	17 (13.9)	
PIK3CA mutational status^d^					
WT	182 (67.7)	15 (46.9)	84 (72.4)	83 (68.6)	**0.023**
Mutated	87 (32.3)	17 (53.1)	32 (27.6)	38 (31.4)	

Significant results are displayed in bold.

*NS* not significant.

^a^HPV insertion: EPI episomal, 2J 2 junctions, MJ multiple junctions.

^b^Chi-square test; *p* values for comparison of the EPI group vs. the 2J group vs. the MJ group for each parameter.

^c^Other HPV genotypes: 39, 42, 52, 56, 58, 59, 68, 70, 73, and 82.

^d^Information available for 269 patients.

Interestingly, coinfections were observed in 12 CC patients (Supplementary Table 2). These tumours presented unique integration site per HPV genotype, where for each case the HPV breakpoints are different.

### Most frequent HPV integration sites

**Fig. 2:**
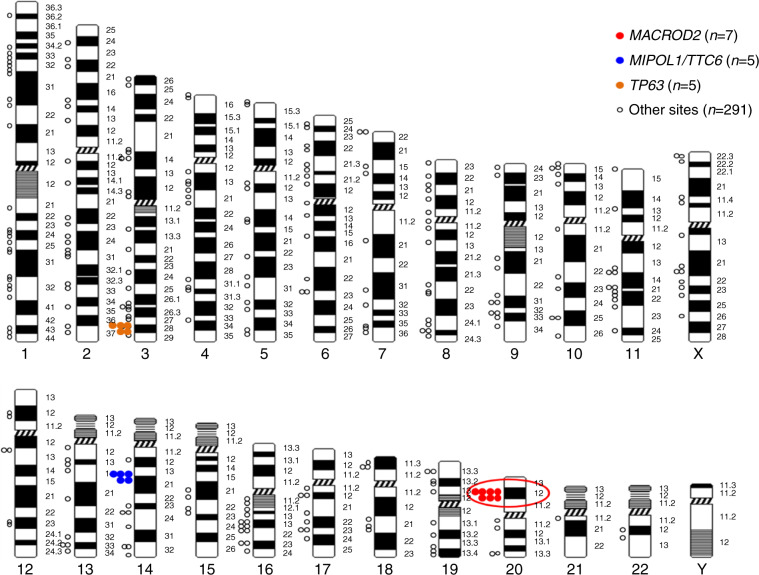
Distribution of HPV insertion sites in the genome of patients with HPV-positive CC. Each dot represents an HPV integration site.

We identified >300 different HPV-chromosomal junctions (inter- or intra-genic) (Fig. 2 and Supplementary Table 3). The most frequent integration site was in the *MACROD2* gene (*n* = 7) (Supplementary Fig. 2) followed by the *MIPOL1/TTC6* (*n* = 5), *TP63* (*n* = 5), and several others such as *ERBB2* (two sites); *KLF12*, and *RAD51B* with a single site (Fig. 2 and Supplementary Table 3). The two tumours with *ERBB2* integration sites were whole-exome sequenced and both showed *ERBB2* amplifications.^15^

### Association between HPV insertion mechanisms with clinical and biological parameters

The distribution of HPV integration signatures according to clinical, biological and pathological characteristics is presented in Table 2 and Supplementary Table 1. While episomal forms were more frequent in *PIK3CA* mutated tumours (*p* = 0.023), HPV integration signatures were not associated with histological subtype, with FIGO stage/lymph nodes (presently FIGO stage 3), or treatment assignation but they were associated with HPV genotype status (*p* < 0.0001). HPV18 and HPV45 genotypes were always integrated (most frequently as 2J). Multiple (MJ) viral integration signatures were predominant in HPV16-positive samples (*n* = 86/155, 57%) as compared to other HPV genotypes (*n* = 36/117; 31%) (Table 2; *p* < 0.0001).

### Association between the insertion mechanisms and the progression-free survival

There was no significant correlation between the HPV integration signatures (EPI, 2J and MJ) and the PFS (Supplementary Fig. 3a, 3b). Similarly, there was no significant association between the HPV integration signatures and the PFS in the subgroup of HPV16-positive patients (data not shown).

The most frequent integration site was in the *MACROD2* gene (*n* = 7) (Supplementary Fig. 2). Patients with HPV integration sites into the *MACROD2* gene (introns 5, 6 and 7) did not have a significantly poorer outcome but the numbers are insufficient to draw any conclusions (*p* = 0.38, Supplementary Fig. 3c). In an exploratory study, interestingly, patients harbouring several viral types did not seem to do worse as compared to patients with single viral infections (Supplementary Fig. 3d), but this did not reach statistical significance (*p* = 0.09).

### Comparison of HPV copy number to HPV subtypes, insertion patterns and outcome

**Fig. 3:**
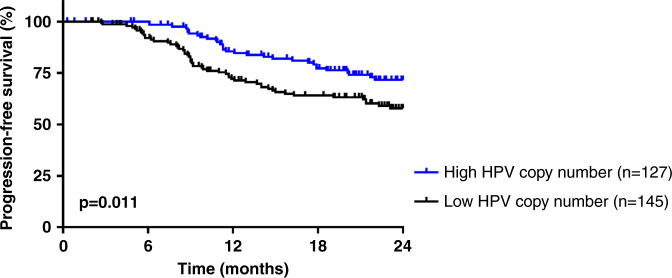
Progression-free survival of the 272 HPV-positive cervical cancer patients according to HPV copy number.

The HPV copy number was estimated by the ratio of the number of HPV reads over the control human gene *KLK3*. The optimal cut-off was four (as determined in the “Methods” section). Patients were classified into low (ratio < 4, *n* = 145) vs. high HPV copy number (ratio ≥ 4, *n* = 127). HPV16-positive patients consistently had a higher HPV copy number (*n* = 95/155, 61%) (*p* < 0.0001) as compared to patients with other HPV subtypes (*n* = 32/117, 27%) (Table 3). Samples with 2J type insertions displayed a low HPV copy number while MJ type insertions were associated with a high HPV copy number (*p* < 0.0001). Furthermore, patients with a low HPV copy number showed poor outcome in comparison to patients with a high HPV copy number (*p* = 0.011) (Fig. 3).

**Table 3:** Clinical and biological characteristics of 272 HPV-positive cervical cancer, in relation to HPV copy number.

		Number of patients (%)		
HPV copy number^a^	Patients (%)	Low HPV copy number (<4)	High HPV copy number (≥4)	*p* value^b^
Total	272 (100.0)	145	127	
Age				
≤50	140 (51.5)	72 (49.7)	68 (53.5)	0.52 (NS)
>50	132 (48.5)	73 (50.3)	59 (46.5)	
Histologic subtype				
Squamous cell carcinoma	230 (84.6)	116 (80.0)	114 (89.8)	0.14 (NS)
Adenocarcinoma	27 (9.9)	19 (13.1)	8 (6.3)	
Adenosquamous carcinoma	10 (3.7)	6 (4.1)	4 (3.1)	
Mixed form or undifferenciated	5 (1.8)	4 (2.8)	1 (0.8)	
HPV status				
Genotype 16	155 (57.0)	60 (41.4)	95 (74.8)	**<0.0001**
Genotype 18	36 (13.2)	28 (19.3)	8 (6.3)	
Genotype 45	27 (9.9)	25 (17.2)	2 (1.6)	
Genotype 31	9 (3.3)	6 (4.1)	3 (2.4)	
Genotype 33	11 (4.0)	4 (2.8)	7 (5.5)	
Other genotypes^c^	34 (12.5)	22 (15.2)	12 (9.4)	
Stage FIGO				
I/II	205 (75.4)	103 (71.0)	102 (80.3)	0.076 (NS)
III/IV	67 (24.6)	42 (29.0)	25 (19.7)	
Lymph node				
Yes	167 (61.4)	101 (69.7)	66 (52.0)	**0.0028**
No	105 (38.6)	44 (30.3)	61 (48.0)	
Pelvis lymph node				
Yes	165 (60.7)	100 (69.0)	65 (51.2)	**0.0027**
No	107 (39.3)	45 (31.0)	62 (48.8)	
Para-aortic lymph node				
Yes	43 (15.8)	28 (19.3)	15 (11.8)	0.091 (NS)
No	229 (84.2)	117 (80.7)	112 (88.2)	
Initial therapy				
Surgery	54 (19.9)	30 (20.7)	24 (18.9)	0.58 (NS)
Radiotherapy	176 (64.7)	90 (62.1)	86 (67.7)	
Neoadjuvant chemotherapy	42 (15.4)	25 (17.2)	17 (13.4)	
PIK3CA mutational status^d^				
WT	182 (67.7)	101 (71.1)	81 (63.8)	0.20 (NS)
Mutated	87 (32.3)	41 (28.9)	46 (36.2)	
HPV insertion^e^				
EPI	33 (12.1)	9 (6.2)	24 (18.9)	**<0.0001**
2J	117 (43.0)	104 (71.7)	13 (10.2)	
MJ	122 (48.9)	32 (22.1)	90 (70.9)	

Significant results are displayed in bold

*NS* not significant.

^a^HPV copy number is a ratio of the number of reads of HPV over control human gene KLK3.

^b^Chi-square test.

^c^Other genotypes.

^d^Information available for 269 patients.

^e^HPV insertion: EPI episomal, 2J 2 junctions, MJ multiple junctions.

## Discussion

In this CC patient population from the prospective BioRAIDs study, we were able to identify >300 HPV-chromosomal (inter-genic or intra-genic) junctions; the *MACROD2* gene being the most frequent integration site (*n* = 7), followed by *MIPOL1/TTC6* (*n* = 5) and *TP63* (*n* = 5). Interestingly, our data identified a new CC-related recurrent integration site in the *MACROD2* (mono-ADP-ribosylhydrolase) gene. Non-coding and structural mutations/variations in the germline *MACROD2* gene have been associated with psychiatric disorders, obesity and cancer predisposition.^18–20^ Deletions in the *MACROD2* gene are frequent in colorectal cancer^21,22^ and are reported to alter DNA repair and sensitivity to DNA damage and consequently impact colorectal tumorigenesis.^23^ Neither RNA expression nor functional studies support a tumour suppressor role of *MACROD2* gene. This gene spans more than 2 Mb and constitutes a common fragile site contributing to increased genomic instability.^24,25^ Our results report intronic integration sites in the *MACROD2* gene yet there is still lack of evidence concerning the functional consequence of these intronic integrations within *MACROD2*. Functional analyses are not straightforward due to the high rate of splicing in *MACROD2* and the important number of alternative transcripts (coding and non-coding) of variable size. *MACROD2* deletions and haploinsufficiency were linked to impaired PARP1 activity and chromosomal instability in colorectal cancer^26^ and in liver cancer,^27^ suggesting a tumour suppressing function of this gene. Importantly, the present study identifies HPV integration as a new molecular pattern of *MACROD2* alteration likely causing loss of function, but the seven patients in our cohort with HPV integration in the *MACROD2* gene are presently insufficient to discern a meaningful impact on CC evolution, albeit responsible for genomic instability.

Previously, frequent integrations in other SCCs were reported in the *MYC, TMEM49*, *FANCC* and *RAD51B* genes^28–30^ as well as in the following: *POU5F1B, FHIT, KLF12, KLF5, HMGA2, LRP1B, LEPREL1, DLG2* and *SEMA3D*. Slightly less common integration sites were reported in the following genes: *AGTR2, DMD, CDH7, DCC, HS3ST4, CPNE8, C9orf85, MSX2* and *CADM2.*^9^ Several of these previously reported integration sites into genes such as *FHIT, KLF12, RAD51B* were detected in a single or in two patients of the present CC cohort. HPV integration in *MIPOL1/TTC6* and *TP63* genes were reported in five patients each. Concordant with our results, Parfenov et al. reported in a head and neck squamous cell carcinomas a rearrangement between chromosomes 3 and 13 close to the HPV integration site in a non-coding region but involved in a region of chromosome 3 where *TP63* genes are located.^31^ P63 plays a key role in epidermal keratinocyte proliferation and differentiation and is a master regulator of gene expression pattern and epigenetic landscape that define epidermal fate.^32^ *TP63*-driven enhancer reprogramming promotes aggressive tumour phenotypes in primary pancreatic ductal adenocarcinomas.^33^ HPV integration in *TP63* genes was recently reported in HPV-positive vulvar cancer patients.^34^ In another HPV-positive head and neck squamous cell carcinoma study, HPV sites of integration into *MIPOL1/TTC6* were identified in more than one tumour sample. The integration of HPV into the *ERBB2* gene site was observed in two patients in association with *ERBB2* amplifications, in concordance with previous reports.^10^

Twelve percent of CC patients did not display any HPV integration, while 43% had double junctions and 49% multiple junctions’ signatures. The distribution of HPV signatures in our CC cohort differed from that previously described in HPV-positive anal squamous cell carcinoma with a lower rate of episomal HPV as compared to anal cancer (45%).^17^

No significant association was observed between HPV integration signatures and treatment type, histological subtype or FIGO staging. MJ viral integration signatures were predominant in HPV16-positive samples and tumours with viral integration (2J or MJ) had less frequent activating mutations in *PIK3CA* than those harbouring episomal HPV, confirming previously reported data.^12^ Similar results were also observed when considering only HPV16 patients (data not shown). This is in accordance with the literature where HPV integration is reported to provide a selective growth advantage of cancer cells.^6^ CC patients with a high HPV copy number had significantly better PFS, as compared to patients with low HPV copy number. These results are consistent with other reports in the literature.^35,36^

In conclusion, while HPV integration is thought to be a random event, our results point out that some hotspots may impact cancer evolution. This would need analyses in larger aggregated datasets. The episomal form of HPV was less frequent in cervical carcinoma as compared to another genital carcinoma (anal carcinoma) and its presence was significantly associated with high HPV copy number, suggesting a decrease of viral replication upon integration. Mutations in *PIK3CA* were significantly associated with high HPV copy number and with the episomal form of HPV. The analysis of outcome based on *PIK3CA* alone did not show an association with poor outcome. In a prior analysis of the BioRAIDS dataset, the association of *PIK3CA* with epigenetic alterations was associated with a shorter PFS.^15^

To our knowledge, this is the first study assessing the prognostic value of HPV integration in a prospectively annotated patient cohort and reporting an HPV integration at the *MACROD2* gene, known to be implicated in impaired PARP1 activity and chromosome instability.

## Supplementary information

Supplementary file

## Data Availability

Clinical and whole-exome sequencing data related to the BioRAIDs patients were previously published in Scholl et al.^15^

## References

[1] Ferlay J, Soerjomataram I, Dikshit R, Eser S, Mathers C, Rebelo M (2015). Cancer incidence and mortality worldwide: sources, methods and major patterns in GLOBOCAN (2012). Int. J. Cancer.

[2] Schiffman MH, Bauer HM, Hoover RN, Glass AG, Cadell DM, Rush BB (1993). Epidemiologic evidence showing that human papillomavirus infection causes most cervical intraepithelial neoplasia. J. Natl Cancer Inst..

[3] Wentzensen N, Vinokurova S, von Knebel Doeberitz M (2004). Systematic review of genomic integration sites of human papillomavirus genomes in epithelial dysplasia and invasive cancer of the female lower genital tract. Cancer Res..

[4] Crosbie EJ, Einstein MH, Franceschi S, Kitchener HC (2013). Human papillomavirus and cervical cancer. Lancet.

[5] Oyervides-Muñoz MA, Pérez-Maya AA, Rodríguez-Gutiérrez HF, Gómez-Macias GS, Fajardo-Ramírez OR, Treviño V (2018). Understanding the HPV integration and its progression to cervical cancer. Infect. Genet. Evol..

[6] Rusan M, Li YY, Hammerman PS (2015). Genomic landscape of human papillomavirus-associated cancers. Clin. Cancer Res..

[7] Xu F, Cao M, Shi Q, Chen H, Wang Y, Li X (2015). Integration of the full-length HPV16 genome in cervical cancer and Caski and Siha cell lines and the possible ways of HPV integration. Virus Genes.

[8] Akagi K, Li J, Broutian TR, Padilla-Nash H, Xiao W, Jiang B (2014). Genome-wide analysis of HPV integration in human cancers reveals recurrent, focal genomic instability. Genome Res..

[9] Hu Z, Zhu D, Wang W, Li W, Jia W, Zeng X (2015). Genome-wide profiling of HPV integration in cervical cancer identifies clustered genomic hot spots and a potential microhomology-mediated integration mechanism. Nat. Genet..

[10] Ojesina AI, Lichtenstein L, Freeman SS, Pedamallu CS, Imaz-Rosshandler I, Pugh TJ (2014). Landscape of genomic alterations in cervical carcinomas. Nature.

[11] McBride AA, Warburton A (2017). The role of integration in oncogenic progression of HPV-associated cancers. PLoS Pathog..

[12] Holmes A, Lameiras S, Jeannot E, Marie Y, Castera L, Sastre-Garau X (2016). Mechanistic signatures of HPV insertions in cervical carcinomas. NPJ Genom. Med..

[13] Samuels S, Balint B, von der Leyen H, Hupé P, de Koning L, Kamoun C (2016). Precision medicine in cancer: challenges and recommendations from an EU-funded cervical cancer biobanking study. Br. J. Cancer.

[14] Ngo C, Samuels S, Bagrintseva K, Slocker A, Hupé P, Kenter G (2015). From prospective biobanking to precision medicine: BIO-RAIDs—an EU study protocol in cervical cancer. BMC Cancer.

[15] Scholl S, Popovic M, de la Rochefordiere A, Girard E, Dureau S, Mandic A (2019). Clinical and genetic landscape of treatment naive cervical cancer: alterations in PIK3CA and in epigenetic modulators associated with sub-optimal outcome. EBioMedicine.

[16] Hanley JA, McNeil BJ (1982). The meaning and use of the area under a receiver operating characteristic (ROC) curve. Radiology.

[17] Morel A, Neuzillet C, Wack M, Lameiras S, Vacher S, Deloger M (2019). Mechanistic signatures of human papillomavirus insertions in anal squamous cell carcinomas. Cancers (Basel).

[18] Lo ReO, Mazza T, Vinciguerra M (2018). Mono-ADP-ribosylhydrolase MACROD2 is dispensable for murine responses to metabolic and genotoxic insults. Front. Genet..

[19] Lombardo B, Esposito D, Iossa S, Vitale A, Verdesca F, Perrotta C (2019). Intragenic deletion in MACROD2: a family with complex phenotypes including microcephaly, intellectual disability, polydactyly, renal and pancreatic malformations. Cytogenet. Genome Res..

[20] Hu N, Kadota M, Liu H, Abnet CC, Su H, Wu H (2016). Genomic landscape of somatic alterations in esophageal squamous cell carcinoma and gastric cancer. Cancer Res..

[21] Cancer Genome Atlas Network. (2012). Comprehensive molecular characterization of human colon and rectal cancer. Nature.

[22] Andersen CL, Lamy P, Thorsen K, Kjeldsen E, Wikman F, Villesen P (2011). Frequent genomic loss at chr16p13.2 is associated with poor prognosis in colorectal cancer. Int. J. Cancer.

[23] Jin N, Burkard ME (2018). MACROD2, an original cause of CIN?. Cancer Discov..

[24] Fungtammasan A, Walsh E, Chiaromonte F, Eckert KA, Makova KD (2012). A genome-wide analysis of common fragile sites: what features determine chromosomal instability in the human genome?. Genome Res..

[25] Feijs KLH, Cooper CDO, Žaja R (2020). The controversial roles of ADP-ribosyl hydrolases MACROD1, MACROD2 and TARG1 in carcinogenesis. Cancers (Basel).

[26] Sakthianandeswaren A, Parsons MJ, Mouradov D, MacKinnon RN, Catimel B, Liu S (2018). MACROD2 haploinsufficiency impairs catalytic activity of PARP1 and promotes chromosome instability and growth of intestinal tumors. Cancer Discov..

[27] Fujimoto A, Furuta M, Totoki Y, Tsunoda T, Kato M, Shiraishi Y (2016). Whole-genome mutational landscape and characterization of noncoding and structural mutations in liver cancer. Nat. Genet..

[28] Zhang R, Shen C, Zhao L, Wang J, McCrae M, Chen X (2016). Dysregulation of host cellular genes targeted by human papillomavirus (HPV) integration contributes to HPV-related cervical carcinogenesis. Int. J. Cancer.

[29] Zhang Y, Koneva LA, Virani S, Arthur AE, Virani A, Hall PB (2016). Subtypes of HPV-positive head and neck cancers are associated with HPV characteristics, copy number alterations, PIK3CA mutation, and pathway signatures. Clin. Cancer Res..

[30] Koneva LA, Zhang Y, Virani S, Hall PB, McHugh JB, Chepeha DB (2018). HPV integration in HNSCC correlates with survival outcomes, immune response signatures, and candidate drivers. Mol. Cancer Res..

[31] Parfenov M, Pedamallu CS, Gehlenborg N, Freeman SS, Danilova L, Bristow CA (2014). Characterization of HPV and host genome interactions in primary head and neck cancers. Proc. Natl Acad. Sci. USA.

[32] Soares E, Zhou H (2018). Master regulatory role of p63 in epidermal development and disease. Cell Mol. Life Sci..

[33] Somerville TDD, Xu Y, Miyabayashi K, Tiriac H, Cleary CR, Maia-Silva D (2018). TP63-mediated enhancer reprogramming drives the squamous subtype of pancreatic ductal adenocarcinoma. Cell Rep..

[34] Thomas J, Leufflen L, Chesnais V, Diry S, Demange J, Depardieu C (2020). Identification of specific tumor markers in vulvar carcinoma through extensive human papillomavirus DNA characterization using next generation sequencing method. J. Low Genit. Trac. Dis..

[35] Deng T, Feng Y, Zheng J, Hg Q, Liu J (2015). Low initial human papillomavirus viral load may, indicate worse proguannosis in patients with cervical carcinoma treated with surgery. J. Gynecol. Oncol..

[36] Lei J, Ploner A, Lagheden C, Eklund C, Nordqvist Kleppe S, Andrae B (2018). High-risk human papillomavirus status and prognosis in invasive cervical cancer: a nationwide cohort study. PLoS Med..

