# Correction to: Human papilloma virus (HPV) integration signature in Cervical Cancer: identification of *MACROD2* gene as HPV hot spot integration site

**DOI:** 10.1038/s41416-023-02261-7

**Published:** 2023-04-13

**Authors:** Maud Kamal, Sonia Lameiras, Marc Deloger, Adeline Morel, Sophie Vacher, Charlotte Lecerf, Célia Dupain, Emmanuelle Jeannot, Elodie Girard, Sylvain Baulande, Coraline Dubot, Gemma Kenter, Ekaterina S. Jordanova, Els M. J. J. Berns, Guillaume Bataillon, Marina Popovic, Roman Rouzier, Wulfran Cacheux, Christophe Le Tourneau, Alain Nicolas, Nicolas Servant, Suzy M. Scholl, Ivan Bièche, RAIDs Consortium, Anne de la Rochefordiere, Pierre Fumoleau, Aljosa Mandic, Nina Samet, Choumouss Kamoun, Windy Rondoff, Sebastien Armanet, Alexandra Rohel, Souhir Neffati, Marie-Emmanuelle Legrier, Sinette Ngoumou Mabiala, Sylvain Dureau, Coralie Errera, Marius Craina, Madalin Margan, Sanne Samuels, Henry Zijlmans, Peter Hillemanns, Sorin Dema, Alis Dema, Goran Malenkovic, Branislav Djuran, Anne Floquet, Frédéric Guyon, Pierre Emmanuel Colombo, Michel Fabbro, Christine Kerr, Charlotte Ngo, Fabrice Lecuru, Eleonor Rivin del Campo, Charles Coutant, Frédéric Marchal, Nathalie Mesgouez-Nebout, Virginie Fourchotte, Jean Guillaume Feron, Philippe Morice, Eric Deutsch, Pauline Wimberger, Jean-Marc Classe, Heiko von der Leyen, Mathieu Minsat, Istvan Nagy, Balazs Balint, Nicolas de Saint-Jorre, Alexia Savignoni, Franck Perez, Patricia Tresca, Noreen Gleeson, Philippe Hupe, Sergio Roman Roman, Emmanuel Barillot, Fanny Coffin, Bastiaan Nuijen, Alexandre Boissonnas, Marc Billaud, Laurence Lafanechere, Jaap Verweij, Arjan Bandel, Jozien Hellemann, Kirsten Ruigrok-Ritstier, Philipp Harter, Christian Kurzeder, Alexander Mustea, Eugeniu Banu, Elisabeta Patcas, Victor Cernat, Andrea Slocker, Michele Mondini, Maud Bossard, Julie Chupin, Sjoerd Rodenhuis, Rene Medema, Anika Havemeier, Thomas Fink, Amelie Michon, Christine Kubiak, Corine Beaufort, Judit Cseklye, Dora Latinovics, Peter Bihari, Isabel Brito, Bérengère Ouine, Leanne De Koning, Vincent Puard, Elaine Del Nery, Jos Beijnen, Dominique Koensgen, Daniela Bruennert, Milos Lucic, Natalja ter Haar

**Affiliations:** 1 Department of Drug Development and Innovation, Institut Curie, PSL Research University, 75005 Paris & 92210, Saint-Cloud, France; 2 Department of Drug Development and Innovation, Institut Curie, PSL Research University, 92210 Saint-Cloud, France; 3 Institut Curie, Genomics of Excellence (ICGex) Platform, PSL Research University, 75005 Paris, France; 4 Bioinformatics and Computational Systems Biology of Cancer, PSL Research University, Mines Paris Tech, INSERM U900, 75005 Paris, France; 5 Department of Genetics, Institut Curie, PSL Research University, 75005 Paris, France; 6 Department of Pathology, Institut Curie, PSL Research University, 75005 Paris, France; 7 Center for Gynaecologic Oncology Amsterdam, Amsterdam UMC and The Netherlands Cancer Institute - Antoni van Leeuwenhoek Hospital, Amsterdam, The Netherlands; 8 Department of Pathology, Leiden University Medical Center, Leiden, The Netherlands; 9 Department of Medical Oncology, Erasmus MC, 3000 CA Rotterdam, The Netherlands; 10 Oncology Institute of Vojvodina, Put doktora Goldmana, 421204 Sremska Kamenica, Serbia; 11 Department of Surgery, Institut Curie, PSL Research University, 92210 Saint-Cloud, France; 12 Paris-Saclay University, Paris, France; 13 Hopital Privé Pays de Savoie, Service d’oncologie médicale, 19 avenue Pierre Mendès France, 74100 Annemasse, France; 14 Institut Curie, PSL Research University, CNRS UMR3244, 75248 Paris, France; 15 Faculty of Pharmaceutical and Biological Sciences, INSERM U1016, Paris Descartes University, 75005 Paris, France; 16 Department of Drug Development and Innovation, Institut Curie, PSL Research University, Paris, France; 17 Institut Curie, Paris, France; 18 Gynecologic Oncology Department Clinic for Operative Oncology, Institute of Oncology of Vojvodina, Faculty of Medicine Novi Sad, University of Novi Sad, Serbia, Sremska, Serbia; 19 Publica Institutul Oncologic, Chișinău, Republic of Moldova; 20 Gustave Roussy, Paris, France; 21 Arcagy-Gineco, Paris, France; 22 Insitut Pasteur, Paris, France; 23 Quanticsoft, Paris, France; 24 University of Medicine and Pharmacy “Victor Babeș”, Timișoara, Romania; 25 Center for Gynaecologic Oncology Amsterdam, Amsterdam UMC and The Netherlands Cancer Institute - Antoni van Leeuwenhoek Hospital, Amsterdam, Netherlands; 26 Hannover Medical School, Hanover, Germany; 27 Chirurgie onco-gynécologique and Oncology, Institut Bergonié, Centre Régional de Lutte contre le Cancer Bordeaux-Aquitaine, Bordeaux, France; 28 Centre Val d’Aurelle, Paris, France; 29 Service de chirurgie cancérologique gynécologique et du sein, Hôpital Européen Georges Pompidou, APHP et faculté de médecine, Université Paris Descartes, Paris, France; 30 Department of Surgery, Institut Curie, PSL Research University, 75005 Paris, France; 31 Department of Surgery, Institut Curie, PSL Research University, 92210 Saint-Cloud, France; 32 Department of Radiation Oncology, Tenon University Hospital, Hôpitaux Universitaires Est Parisien, Sorbonne University Medical Faculty, Paris, France; 33 Centre Georges François Leclerc, Paris, France; 34 Département de chirurgie, CRAN, UMR 7039, Université de Lorraine, CNRS, Institut de Cancérologie de Lorraine, Vandœuvre-lès-Nancy, France; 35 Institut de cancérologie de l’Ouest - site Paul Papin (ICO), Paris, France; 36 Department of Gynecology and Obstetrics, Universitätsklinikum Carl Gustav Carus; an der Technischen Universität Dresden, Fetscherstraße 74, 01307 Dresden, Germany; 37 René Gauducheau, Paris, France; 38 Hannover Clinical Trial Center GmbH, Hannover, Germany; 39 SeqOmics Biotechnology Ltd, Vallalkozok utja 7, Morahalom, Hungary; 40 Institut Curie, PSL Research University, Paris, France; 41 Insitut Curie, Paris, France; 42 St James/Trinity College, Dublin, Ireland; 43 Netherlands Cancer Institute, Amsterdam, The Netherlands; 44 Université Pierre et Marie Curie, Paris, France; 45 Université Joseph-Fourier, Grenoble, France; 46 Erasmus University Medical Centre, Rotterdam, Netherlands; 47 Department of Medical Oncology, Erasmus MC Cancer Institute, Erasmus University Medical Center, 3015 CN Rotterdam, Netherlands; 48 Department of Gynecology and Gynecologic Oncology, Ev. Kliniken Essen-Mitte, Essen, Germany; 49 Department of Obstetrics and Gynecology, University Hospital of Basel, Basel, Switzerland; 50 Department of Gynecology and Gynecological Oncology, University Hospital, 53127 Bonn, Germany; 51 Spitalul Sfantul Constantin Brasov, Brasov, Romania; 52 Department of Gynecology and Obstetrics, University Medicine Greifswald, Greifswald, Germany; 53 Ayming, Gennevilliers, France; 54 European Clinical Research Infrastructure Network, Toulouse, France; 55 University of Greifswald, Greifswald, Germany; 56 Oncology Institute of Vojvodina, Diagnostic Imaging Centre, University of Novi Sad, University School of Medicine, Put Dr. Goldmana 4, Sremska Kamenica, 21204 Novi Sad, Serbia

**Keywords:** Oncology, Molecular medicine, Biomarkers, Molecular biology

Correction to: *British Journal of Cancer* 10.1038/s41416-020-01153-4, published online 16 November 2020

The original version of this article contained an error in an affiliation. Upon careful review of the paper, the authors noticed that the affiliation for Goran Malenkovic was not written correctly. The authors only submitted the following affiliation Gynecologic Oncology Department Clinic for Operative Oncology, Institute of Oncology of Vojvodina, Serbia but they missed to notice that the Faculty of Medicine Novi Sad, University of Novi Sad, Serbia should also be in the affiliation.

