# Supplementary material for: Human papilloma virus (HPV) integration signature in Cervical Cancer: identification of MACROD2 gene as HPV hot spot integration site
Source: Br J Cancer. 2020 Nov 16;124(4):777–85. doi: 10.1038/s41416-020-01153-4 (PMC7884736; doi:10.1038/s41416-020-01153-4)
Supplement: Supplementary file 1 — Supplementary file [file 41416_2020_1153_MOESM1_ESM.pdf]

Supplementary Figure 1: Distribution of the HPV integration signatures according to the location of HPV16-positive squamous cell carcinomas: a: cervical cancer; b: anal cancer; p= 0.0003.

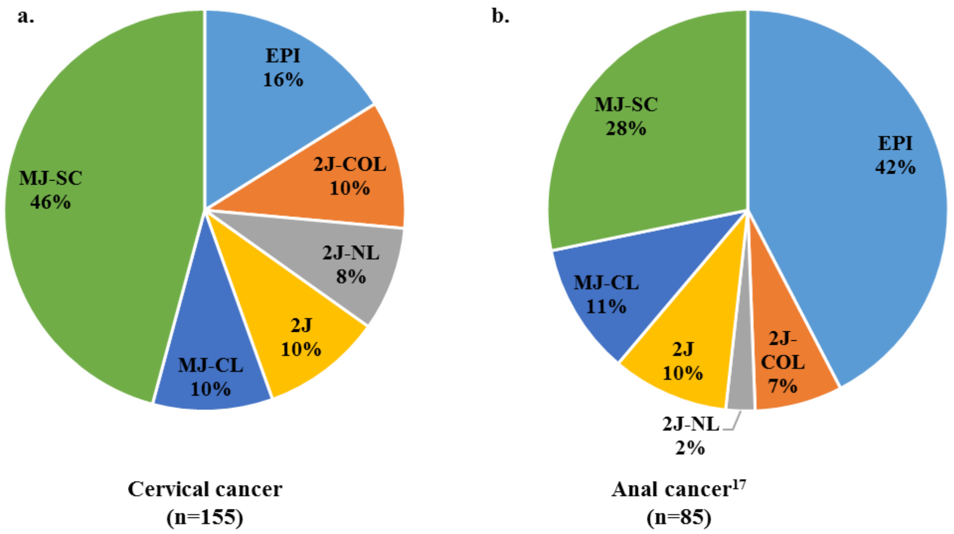

Supplementary Figure 2: HPV integration sites in the *MACROD2* gene (GRCh37 / hg19)

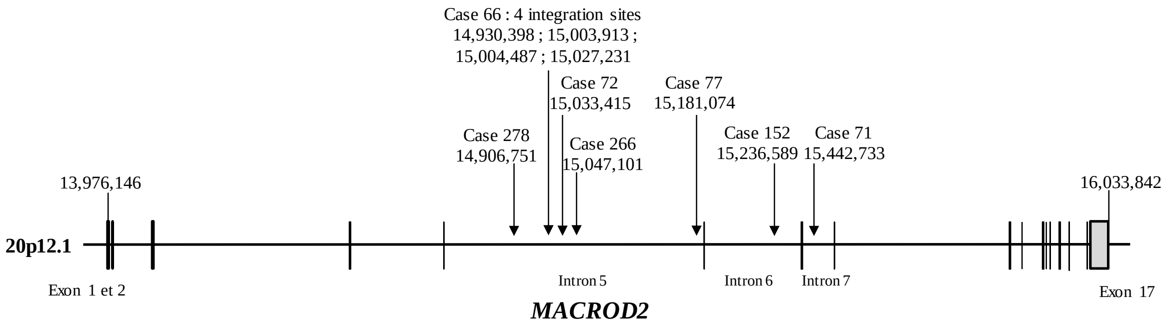

Supplementary Figure 3: Progression-free survival of the 272 HPV-positive cervical cancer patients according to HPV integration signatures (a, b), to (c) HPV integration into *MACROD2* and to (d) coinfection.

a.

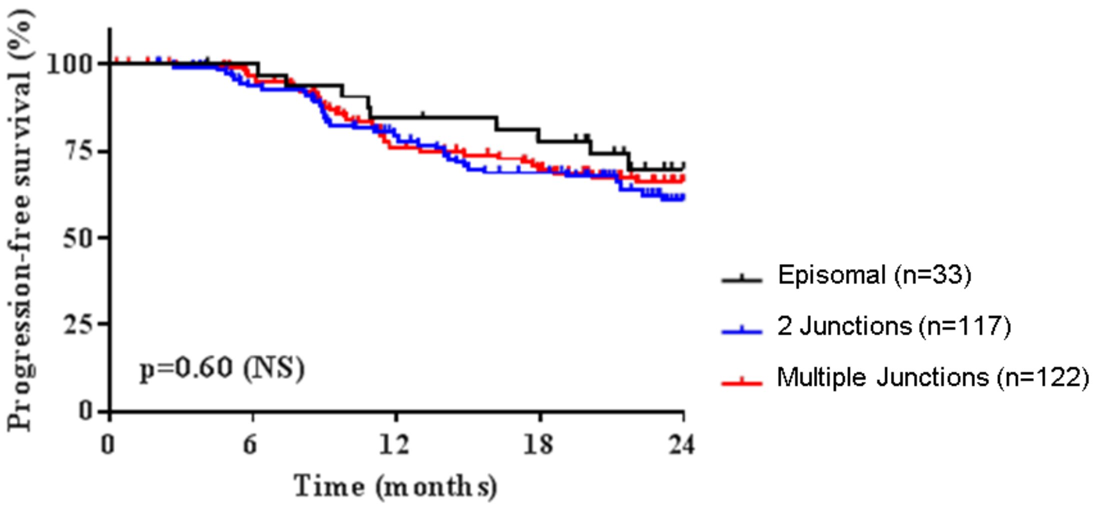

b.

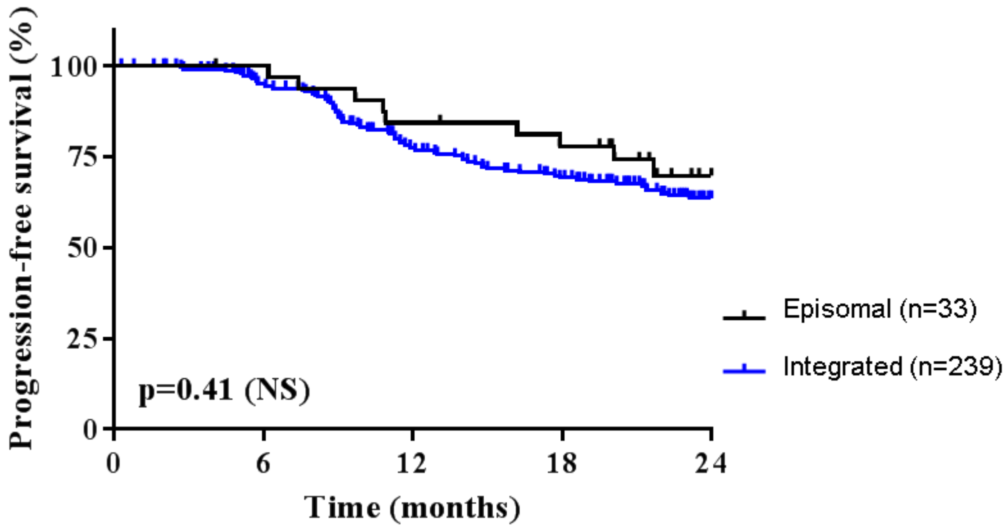

c.

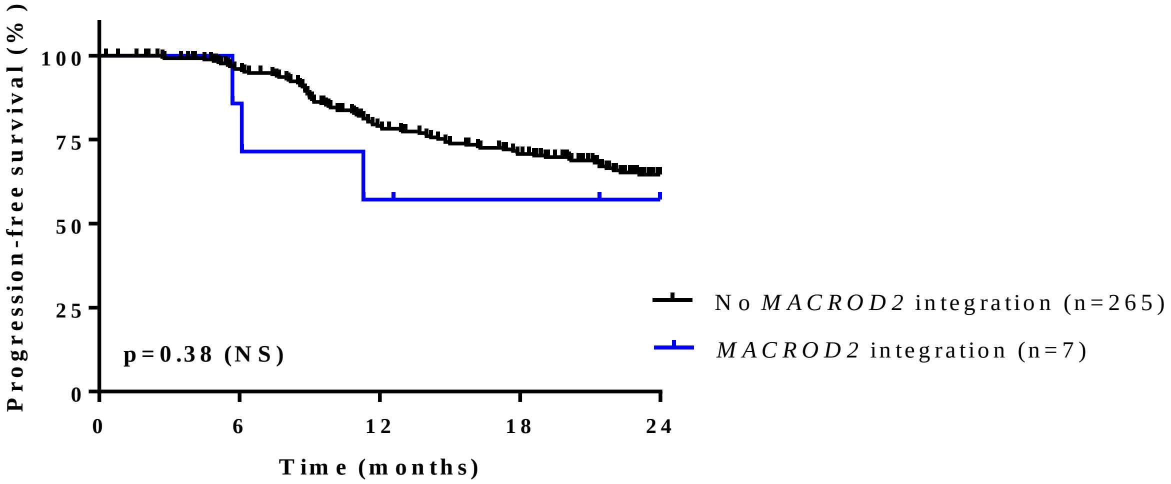

Progression-free survival (%)
100
75
50
25
0
0
6
12
18
24
Time (months)
p=0.38 (NS)
No *MACROD2* integration (n=265)
MACROD2 integration (n=7)

d.

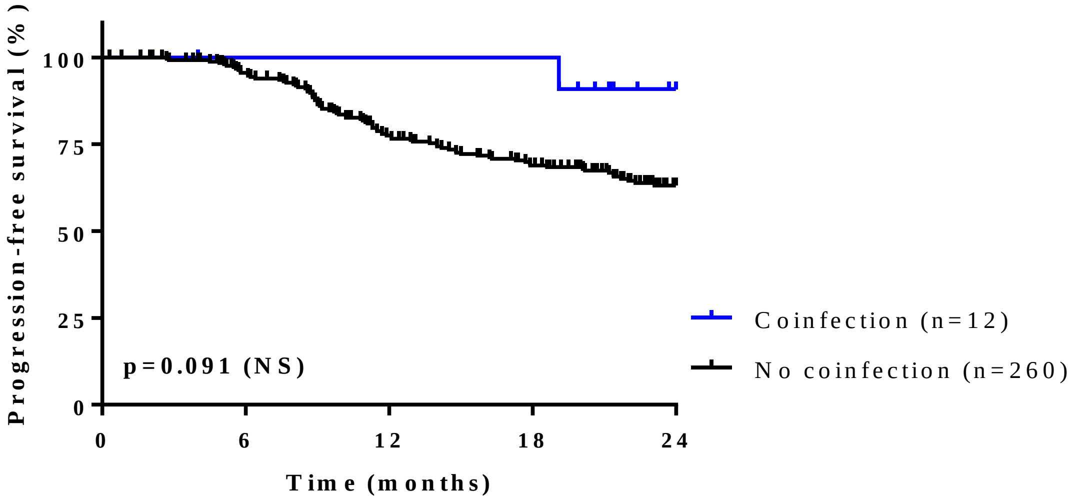

Progression-free survival (%)
100
75
50
25
0
0
6
12
18
24
Time (months)
p=0.091 (NS)
Coinfection (n=12)
No coinfection (n=260)

Supplementary Table 1: Relationship between mechanisms of integration of HPV and clinical, biological and pathological characteristics of the 272 patients with HPV-positive cervical cancer

	**Patients (%)**	**Number of patients (%)**						***p*-value**[b]
**HPV insertion**[a]		***EPI***	***2J-COL***	***2J-NL***	***2J***	***MJ-CL***	***MJ-SC***	
Total	272 (100·0)	33 (12·1)	30 (11·0)	53 (19·5)	34 (12·5)	27 (9·9)	95 (34·9)	
Age								
≤50	140 (51·5)	11 (33·3)	14 (46·7)	30 (56·6)	16 (47·1)	14 (51·9)	55 (57·9)	0·22 (NS)
>50	132 (48·5)	22 (66·7)	16 (53·3)	23 (43·4)	18 (52·9)	13 (48·1)	40 (42·1)	
Histologic subtype								
Squamous cell carcinoma	230 (84·6)	27 (81·8)	26 (86·1)	39 (73·6)	27 (79·4)	25 (92·6)	86 (90·5)	0·14 (NS)
Adenocarcinoma	27 (9·9)	3 (9·1)	3 (10·0)	12 (22·6)	3 (8·8)	1 (3·7)	5 (5·3)	
Adenosquamous carcinoma	10 (3·7)	2 (6·1)	0 (0·0)	2 (3·8)	2 (5·9)	1 (3·7)	3 (3·2)	
Mixed form or undifferenciated	5 (1·8)	1 (3·0)	1 (3·3)	0 (0·0)	2 (5·9)	0 (0·0)	1 (1·1)	
HPV Status								
Genotype 16	155 (57·0)	25 (75·8)	16 (53·3)	13 (24·5)	15 (44·1)	15 (55·6)	71 (74·7)	**<0·0001**
Genotype 18	36 (13·2)	0 (0·0)	6 (20·0)	14 (26·4)	4 (11·8)	4 (14·8)	8 (8·4)	
Genotype 45	27 (9·9)	0 (0·0)	3 (10·0)	16 (30·2)	5 (14·7)	2 (7·4)	1 (1·1)	
Genotype 31	9 (3·3)	2 (6·1)	1 (3·3)	1 (1·9)	2 (5·9)	2 (7·4)	1 (1·1)	
Genotype 33	11 (4·0)	3 (9·1)	0 (0·0)	2 (3·8)	2 (5·9)	1 (3·7)	3 (3·2)	
Other genotypes [c]	34 (12·5)	3 (9·1)	4 (13·3)	7 (13·2)	6 (17·6)	3 (11·1)	11 (11·6)	
Stage FIGO								
I/II	205 (75·4)	25 (75·8)	23 (76·7)	38 (71·7)	25 (73·5)	18 (66·7)	76 (80·0)	0·75 (NS)
III/IV	67 (24·6)	8 (24·2)	7 (23·3)	15 (28·3)	9 (26·5)	9 (33·3)	19 (20·0)	
Lymph node								
Yes	167 (61·4)	19 (57·6)	17 (56·7)	41 (77·4)	23 (67·6)	17 (63·0)	50 (52·6)	0·080 (NS)
No	105 (38·6)	14 (42·4)	13 (43·3)	12 (22·6)	11 (32·4)	10 (37·0)	45 (47·4)	
Pelvis lymph node								
Yes	165 (60·7)	19 (57·6)	17 (56·7)	40 (75·5)	23 (67·6)	17 (63·0)	49 (51·6)	0·10 (NS)
No	107 (39·3)	14 (42·4)	13 (43·3)	13 (24·5)	11 (32·4)	10 (37·0)	46 (48·4)	
Para-aortic lymph node								
Yes	43 (15·8)	3 (9·1)	2 (6·7)	11 (20·8)	7 (20·6)	5 (18·5)	15 (15·8)	0·45 (NS)
No	229 (84·2)	30 (90·9)	28 (93·3)	42 (79·2)	27 (79·4)	22 (81·5)	80 (84·2)	
Initial Therapy								
Surgery	54 (19·9)	7 (21·2)	4 (13·3)	8 (15·1)	9 (26·5)	8 (29·6)	18 (18·9)	0·34 (NS)
Radiotherapy	176 (64·7)	22 (66·7)	24 (80·0)	34 (64·2)	17 (50·0)	14 (51·9)	65 (68·4)	
Neoadjuvant chemotherapy	42 (15·4)	4 (12·1)	2 (6·7)	11 (20·8)	8 (23·5)	5 (18·5)	12 (12·6)	
PIK3CA Mutational Status [d]								
WT	182 (67·7)	15 (46·9)	22 (73·3)	42 (79·2)	20 (60·6)	18 (66·7)	65 (69·1)	0·054 (NS)
Mutated	87 (32·3)	17 (53·1)	8 (26·7)	11 (20·8)	13 (39·4)	9 (33·3)	29 (30·9)	

[a] HPV insertion**:** 2J :2 junctions, EPI: Episomal, MJ-CL: Multiple Junctions Clustered, MJ-SC: Multiple Junctions Scattered, 2J-NL: 2 Junctions Non-Linear, 2J-COL: 2 Junctions Colinear

[b] Chi-Square Test; *p* values for comparison between EPI, 2J-COL, 2J-NL, 2J, MJ-CL and MJ-SC groups for each parameter

[c] Other genotypes: 39, 42, 52, 56, 58, 59, 68, 70, 73, 82

[d] Information available for 269 patients

NS: not significant. Significant results are displayed in bold

2J: second junction not detected and therefore cannot be classified as co-linear or not

Supplementary Table 2: Coinfections in 12 cervical cancer patients.

Patient	HPV genotype	HPV insertion[a]	HPV reads	
			Number	%
R29	HPV82	2J	96500	95·6
	HPV16	EPI	4400	4·4
R59	HPV33	2J	119702	94·1
	HPV39	MJ-CL	7498	5·9
R60	HPV16	2J-COL	18097	93·0
	HPV33	EPI	1370	7·0
R74	HPV33	EPI	157786	53·7
	HPV35	EPI	104305	35·5
	HPV52	EPI	31519	10·7
R173	HPV18	2J-NL	3217	64·6
	HPV16	2J	1762	35·4
R176	HPV18	2J-NL	2213	63·0
	HPV16	EPI	1301	37·0
R181	HPV73	2J	1185	51·3
	HPV16	EPI	1123	48·7
R195	HPV18	2J-NL	1460	56·2
	HPV16	EPI	1139	43·8
R202	HPV18	2J-COL	909	54·3
	HPV16	EPI	766	45·7
R244	HPV16	MJ-SC	4730	86·4
	HPV18	EPI	747	13·6
R373	HPV58	2J-NL	795	52·7
	HPV16	EPI	714	47·3
R378	HPV16	EPI	935	53·1
	HPV67	2J-COL	827	46·9

[a] HPV insertion**:** 2J :2 junctions, EPI: Episomal, MJ-CL: Multiple Junctions Clustered, MJ-SC: Multiple Junctions Scattered, 2J-NL: 2 Junctions Non-Linear, 2J-COL: 2 Junctions Colinear

Supplementary Table 3: Integration sites in human genes in the 272 cervical cancers

Case No.	HPV genotype	HPV insertion	Chromosomal location	Breakpoint (hg19)	Gene	Position in gene (I/E)	Nb of cases with integration in the gene
**R144**	16	MJ-SC	17q21.33	48722023	*ABCC3*	I	1
**R229**	18	2J-NL	5q31.1	131316287	*ACSL6*	I	1
**R266**	16	MJ-SC	1p33	50349571	*AGBL4*	I	1
**R266**	16	MJ-SC	1p33	50396145	*AGBL4*	I	1
**R266**	16	MJ-SC	1p33	50396204	*AGBL4*	I	1
**R283**	16	MJ-SC	2q31.2	178286280	*AGPS*	I	1
**R89**	16	MJ-SC	1p31.1	77905042	*AK5*	I	1
**R30**	18	2J-NL	14q12	32905741	*AKAP6*	I	1
**R30**	18	2J-NL	14q12	32976351	*AKAP6*	I	1
**R35**	16	MJ-CL	10p15.1	5026685	*AKR1C3*	I	2
**R35**	16	MJ-CL	10p15.1	5081966	*AKR1C3*	I	2
**R224**	16	MJ-SC	10p15.1	5091832	*AKR1C3*	I	2
**R224**	16	MJ-SC	10p15.1	5091834	*AKR1C3*	I	2
**R221**	16	MJ-SC	6p12.3	46720988	*ANKRD66*	I	1
**R41**	16	MJ-SC	4q21.21	80830012	*ANTXR2*	I	1
**R221**	16	MJ-SC	11q22.1	100759627	*ARHGAP42*	I	1
**R33**	16	MJ-SC	Xq26.3	135840140	*ARHGEF6*	I	1
**R169**	16	MJ-SC	Xp11.4	40459008	*ATP6AP2*	I	1
**R41**	16	MJ-SC	10q25.3	117232243	*ATRNL1*	I	2
**R283**	16	MJ-SC	10q25.3	116933741	*ATRNL1*	I	2
**R153**	16	MJ-SC	7p14.3	32931734	*AVL9*	I	1
**R153**	16	MJ-SC	7p14.3	32931688	*AVL9*	I	1
**R136**	16	MJ-SC	8q22.3	103844736	*AZIN1*	I	1
**R342**	82	MJ-CL	16q22.1	67184552	*B3GNT9*	*E*	1
**R253**	16	MJ-SC	14q32.2	99710835	*BCL11B*	I	2
**R253**	16	MJ-SC	14q32.2	99731553	*BCL11B*	I	2
**R253**	16	MJ-SC	14q32.2	99721021	*BCL11B*	I	2
**R347**	16	MJ-SC	14q32.2	99711180	*BCL11B*	I	2
**R347**	16	MJ-SC	14q32.2	99711727	*BCL11B*	I	2
**R347**	16	MJ-SC	14q32.2	99709774	*BCL11B*	I	2
**R347**	16	MJ-SC	14q32.2	99725540	*BCL11B*	I	2
**R238**	18	MJ-SC	13q21.33	68908061	*BMP5*	I	1
**R238**	18	MJ-SC	1q31.1	190285027	*BRINP3*	I	1
**R67**	16	MJ-SC	12q23.3	108026370	*BTBD11*	I	1
**R342**	82	MJ-CL	16q22.1	67161209	*C16Orf70*	I	1
**R342**	82	MJ-CL	16q22.1	67160120	*C16Orf70*	I	1
**R33**	16	MJ-SC	2p24.1	21005899	*C2Orf43*	I	1
**R41**	16	MJ-SC	4q21.1	81571101	*C4Orf22*	I	1
**R253**	16	MJ-SC	5q31.1	134629789	*C5orf66*	I	1
**R253**	16	MJ-SC	5q31.1	134630765	*C5orf66*	I	1
**R253**	16	MJ-SC	5q31.1	134609879	*C5orf66*	I	1
**R41**	16	MJ-SC	6q22.31	121480832	*C6Orf170*	I	1
**R41**	16	MJ-SC	6q22.31	121480842	*C6Orf170*	I	1
**R51**	16	MJ-SC	7p22.3	1150104	*C7Orf50*	I	1
**R51**	16	MJ-SC	7p22.3	1176630	*C7Orf50*	I	1

**R51**	16	MJ-SC	7p22.3	1165643	*C7Orf50*	I	1
**R19**	31	MJ-SC	1q25.1	174980495	*CACYBP*	I	1
**R221**	16	MJ-SC	3p12.1	86054162	*CADM2*	I	1
**R221**	16	MJ-SC	3p12.1	86044157	*CADM2*	I	1
**R221**	16	MJ-SC	3p12.1	86042194	*CADM2*	I	1
**R221**	16	MJ-SC	3p12.1	86022101	*CADM2*	I	1
**R58**	18	MJ-CL	1q32.1	200728911	*CAMSAP2*	I	1
**R58**	18	MJ-CL	1q32.1	200728895	*CAMSAP2*	I	1
**R269**	16	MJ-SC	6p22.2	25360857	*CARMIL1*	I	1
**R187**	16	MJ-SC	8q24.21	128353194	*CASC21,8*	I	1
**R88**	16	2J-NL	8p21.1	27596196	*CCDC25*	I	1
**R393**	16	2J	3p14.3	56603273	*CCDC66*	I	1
**R195**	18	2J-NL	16q22.1	66943209	*CDH16*	E	1
**R47**	18	MJ-SC	8p11.21	42232759	*CEACAM5*	I	2
**R196**	45	2J	19q13.2	42220477	*CEACAM5*	I	2
**R47**	18	MJ-SC	19q13.2	42262206	*CEACAM6*	I	2
**R153**	16	MJ-SC	19q13.2	42267127	*CEACAM6*	I	2
**R41**	16	MJ-SC	10p14	11114858	*CELF2*	I	1
**R41**	16	MJ-SC	10p14	11114814	*CELF2*	I	1
**R41**	16	MJ-SC	10p14	11073508	*CELF2*	I	1
**R41**	16	MJ-SC	10p14	11135737	*CELF2*	I	1
**R180**	16	2J-NL	13q12.12	25484436	*CENPJ*	I	1
**R217**	16	MJ-SC	1q43	243389585	*CEP170*	I	1
**R83**	33	MJ-SC	7q21.13	89878330	*CFAP69*	I	1
**R89**	16	MJ-SC	9q34.13	135412876	*CFAP77*	I	1
**R67**	16	MJ-SC	12q23.3	104938658	*CHST11*	I	1
**R388**	16	MJ-SC	7p22.3	2444851	*CHST2*	I	1
**R388**	16	MJ-SC	7p22.3	2462520	*CHST2*	I	1
**R388**	16	MJ-SC	7p22.3	2466671	*CHST2*	I	1
**R194**	18	2J-NL	3q28	190124524	*CLDN16*	I	1
**R218**	16	MJ-SC	5p15.33	1341546	*CLPTM1L*	I	1
**R140**	16	2J-COL	6p22.3	25102888	*CMAHP*	I	1
**R140**	16	2J-COL	6p22.3	25102895	*CMAHP*	I	1
**R217**	16	MJ-SC	1p22.3	86374874	*COL24A1*	I	1
**R199**	16	MJ-CL	Xq22.3	107903798	*COL4A5*	I	1
**R199**	16	MJ-CL	Xq22.3	107852142	*COL4A5*	I	1
**R253**	16	MJ-SC	5q23.1	115523461	*COMMD10*	I	1
**R89**	16	MJ-SC	8q13.2	68648762	*CPA6*	I	1
**R41**	16	MJ-SC	7p15.1	28580676	*CREB5*	I	1
**R141**	16	MJ-SC	16p13.3	3799335	*CREBBP*	I	2
**R141**	16	MJ-SC	16p13.3	3806127	*CREBBP*	I	2
**R217**	16	MJ-SC	16p13.3	3848213	*CREBBP*	I	2
**R153**	16	MJ-SC	8p23.2	3590903	*CSMD1*	I	1
**R153**	16	MJ-SC	8p23.2	3544846	*CSMD1*	I	1
**R153**	16	MJ-SC	8p23.2	3644222	*CSMD1*	I	1
**R381**	16	MJ-SC	Xq24	119660820	*CUL4B*	I	1
**R168**	45	MJ-CL	4q13.3	74736238	*CXCL1*	I	1
**R168**	45	MJ-CL	4q13.3	74963487	*CXCL2*	I	1
**R59**	39	MJ-CL	5q31.2	139054584	*CXXC5*	I	1
**R59**	39	MJ-CL	5q31.2	139054600	*CXXC5*	I	1

**R363**	16	MJ-CL	1p32.2	57605447	*DAB1*	I	1
**R363**	16	MJ-CL	1p32.2	57551736	*DAB1*	I	1
**R363**	16	MJ-CL	1p32.2	57551724	*DAB1*	I	1
**R283**	16	MJ-SC	14q32.2	100624045	*DEGS2*	I	1
**R283**	16	MJ-SC	14q32.2	100624070	*DEGS2*	I	1
**R223**	45	2J-NL	2q37.1	234369743	*DGKD*	I	1
**R33**	16	MJ-SC	Xq21.33	96479794	*DIAPH2*	I	2
**R137**	16	2J-COL	Xq21.33	96392266	*DIAPH2*	I	2
**R137**	16	2J-COL	Xq21.33	96700438	*DIAPH2*	I	2
**R77**	33	MJ-SC	13q14.2	50669115	*DLEU1*	I	1
**R221**	16	MJ-SC	11q14.1	83964339	*DLG2*	I	1
**R221**	16	MJ-SC	11q14.1	84118459	*DLG2*	I	1
**R153**	16	MJ-SC	18p11.31	3505307	*DLGAP1*	I	1
**R153**	16	MJ-SC	18p11.31	3611314	*DLGAP1*	I	1
**R153**	16	MJ-SC	18p11.31	3533865	*DLGAP1*	I	1
**R153**	16	MJ-SC	18p11.31	3522616	*DLGAP1*	I	1
**R141**	16	MJ-SC	7q36.2	154463449	*DPP6*	I	1
**R89**	16	MJ-SC	1p21.3	97993551	*DPYD*	I	1
**R263**	18	MJ-SC	3q21.3	128005388	*EEFSEC*	I	1
**R236**	16	MJ-SC	11p13	34666305	*EHF*	I	1
**R236**	16	MJ-SC	11p13	34666319	*EHF*	I	1
**R55**	16	MJ-SC	3q27.2	184919754	*EHHADH*	I	1
**R159**	16	MJ-SC	18p11.32	2888958	*EMILIN2*	I	1
**R159**	16	MJ-SC	18p11.32	2879357	*EMILIN2*	I	1
**R68**	16	2J-COL	2q14.2	119605254	*EN1*	E	1
**R68**	16	2J-COL	2q14.2	119605240	*EN1*	E	1
**R159**	16	MJ-SC	13q14.11	44056261	*ENOX1*	I	1
**R133**	16	MJ-SC	1q21.3	150600814	*ENSA*	I	1
**R141**	16	MJ-SC	3p11.1	89468823	*EPHA3*	I	1
**R141**	16	MJ-SC	3p11.1	89336306	*EPHA3*	I	1
**R392**	16	2J-COL	3q27.1	184279512	*EPHB3*	E	1
**R25**	68	MJ-SC	19q13.42	56187256	*EPN1*	I	1
**R67**	16	MJ-SC	17q12	37860015	*ERBB2*	I	2
**R67**	16	MJ-SC	17q12	37860057	*ERBB2*	I	2
**R67**	16	MJ-SC	17q12	37863170	*ERBB2*	I	2
**R67**	16	MJ-SC	17q12	37857248	*ERBB2*	I	2
**R67**	16	MJ-SC	17q12	37857200	*ERBB2*	I	2
**R67**	16	MJ-SC	17q12	37861800	*ERBB2*	I	2
**R67**	16	MJ-SC	17q12	37863166	*ERBB2*	I	2
**R130**	16	MJ-SC	17q12	37847133	*ERBB2*	I	2
**R130**	16	MJ-SC	17q12	37847149	*ERBB2*	I	2
**R72**	16	MJ-SC	3p14.3	55554953	*ERC2*	I	1
**R72**	16	MJ-SC	3p14.3	55573110	*ERC2*	I	1
**R72**	16	MJ-SC	3p14.3	55555137	*ERC2*	I	1
**R72**	16	MJ-SC	3p14.3	55563799	*ERC2*	I	1
**R72**	16	MJ-SC	3p14.3	55563502	*ERC2*	I	1
**R238**	18	MJ-SC	2q31.1	171633284	*ERICH2*	I	1
**R238**	18	MJ-SC	2q31.1	171649381	*ERICH2*	E	1
**R24**	16	MJ-SC	13q13.3	37580726	*EXOSC8*	I	1
**R24**	16	MJ-SC	13q13.3	37580714	*EXOSC9*	I	1

**R325**	16	MJ-SC	15q22.2	59794282	*FAM81A*	I	1
**R340**	16	MJ-CL	7q31.32	121945962	*FEZF1-AS1*	E	1
**R222**	16	MJ-CL	4p16.3	1806219	*FGFR3*	E	1
**R258**	16	2J-NL	3p14.2	61219990	*FHIT*	I	2
**R258**	16	2J-NL	3p14.2	61149007	*FHIT*	I	2
**R269**	16	MJ-SC	3p14.2	60715491	*FHIT*	I	2
**R269**	16	MJ-SC	3p14.2	60760986	*FHIT*	I	2
**R269**	16	MJ-SC	3p14.2	60607350	*FHIT*	I	2
**R144**	16	MJ-SC	3q26.31	171804827	*FNDC3B*	I	1
**R144**	16	MJ-SC	3q26.31	171804890	*FNDC3B*	I	1
**R241**	16	MJ-SC	14q21.1	38060841	*FOXA1*	E	1
**R271**	16	2J-COL	10p13	13739708	*FRMD4A*	I	1
**R271**	16	2J-COL	10p13	13739663	*FRMD4A*	I	1
**R256**	52	MJ-SC	2p22.1	38941523	*GALM*	I	1
**R256**	52	MJ-SC	2p22.1	38941511	*GALM*	I	1
**R66**	16	MJ-SC	7q36.1	151688706	*GALNTL5*	I	1
**R66**	16	MJ-SC	7q36.1	151683314	*GALNTL5*	I	1
**R365**	16	MJ-SC	4q34.1	172894879	*GALNTL6*	I	1
**R153**	16	MJ-SC	18p11.31	3470616	*GAPLINC*	I	1
**R283**	16	MJ-SC	10q25.3	117843626	*GFRA1*	I	1
**R79**	16	MJ-SC	20p13	3641060	*GFRA4*	I	1
**R161**	59	MJ-SC	3p26.1	7518132	*GRM7*	I	1
**R378**	67	2J-COL	16q24.1	85683458	*GSE1*	I	1
**R378**	67	2J-COL	16q24.1	85683434	*GSE1*	I	1
**R342**	82	MJ-CL	16p12.1	28042055	*GSG1L*	I	1
**R342**	82	MJ-CL	16p12.1	28058045	*GSG1L*	I	1
**R272**	45	MJ-SC	Xq13.1	71648549	*HDAC8*	I	1
**R342**	82	MJ-CL	16q22.1	67203167	*HDF4*	E	1
**R161**	59	MJ-SC	14q24.3	73986152	*HEATR4*	I	1
**R285**	18	2J-COL	6p24.1	12104180	*HIVEP1*	I	1
**R285**	18	2J-COL	6p24.1	12104166	*HIVEP1*	I	1
**R144**	16	MJ-SC	5q35.2	173511239	*HMP19*	I	1
**R49**	16	MJ-SC	19p13.2	8523350	*HNRNPM*	I	1
**R49**	16	MJ-SC	19p13.2	8538235	*HNRNPM*	I	1
**R49**	16	MJ-SC	19p13.2	8543437	*HNRNPM*	I	1
**R94**	18	MJ-SC	3p25.3	11186131	*HRH1*	I	1
**R94**	18	MJ-SC	3p25.3	11186153	*HRH1*	I	1
**R342**	82	MJ-CL	16q22.1	67201881	*HSF4*	E	1
**R89**	16	MJ-SC	16p12.1	26025841	*HST3ST4*	I	1
**R197**	18	MJ-SC	9p24.1	6249605	*IL33*	I	1
**R348**	16	MJ-SC	19p13.2	7276589	*INSR*	I	1
**R348**	16	MJ-SC	19p13.2	7287608	*INSR*	I	1
**R216**	16	MJ-SC	16p11.2	30499607	*ITGAL*	I	1
**R244**	16	MJ-SC	19p13.11	17950428	*JAK3*	E	1
**R244**	16	MJ-SC	19p13.11	17950437	*JAK3*	E	1
**R45**	18	2J-COL	6q13	73415545	*KCNQ5*	I	1
**R45**	18	2J-COL	6q13	73441532	*KCNQ5*	I	1
**R249**	16	MJ-SC	6q11.1	62723811	*KHDRBS2*	I	1
**R47**	18	MJ-SC	8q11.21	48339098	*KIAA0146*	I	1
**R47**	18	MJ-SC	8q11.21	48340143	*KIAA0146*	I	1

**R153**	16	MJ-SC	1q44	245823687	*KIF26B*	I	1
**R166**	16	MJ-SC	13q22.1	74522098	*KLF12*	I	1
**R166**	16	MJ-SC	13q22.1	74359968	*KLF12*	I	1
**R269**	16	MJ-SC	1p34.1	44544979	*KLF17*	I	1
**R269**	16	MJ-SC	1p34.1	44548333	*KLF17*	I	1
**R269**	16	MJ-SC	1p34.1	44530015	*KLF17*	I	1
**R269**	16	MJ-SC	1p34.1	44566101	*KLF17*	I	1
**R269**	16	MJ-SC	1p34.1	44554580	*KLF17*	I	1
**R349**	16	MJ-SC	19q13.42	55857051	*KMT5C*	I	1
**R161**	59	MJ-SC	3q28	189725686	*LEPREL1*	I	2
**R161**	59	MJ-SC	3q28	189693489	*LEPREL1*	I	2
**R161**	59	MJ-SC	3q28	189693495	*LEPREL1*	I	2
**R161**	59	MJ-SC	3q28	189725679	*LEPREL1*	I	2
**R250**	16	2J-NL	3q28	189680802	*LEPREL1*	I	2
**R161**	59	MJ-SC	3q28	189736396	*LEPREL2*	I	1
**R269**	16	MJ-SC	19q13.33	48625108	*LIG1*	I	1
**R238**	18	MJ-SC	2p22.3	33141403	*LINC00486*	I	1
**R251**	18	MJ-CL	8q24.21	129454393	*LINC00824*	I	1
**R153**	16	MJ-SC	19q13.12	35905241	*LINC01531*	I	1
**R266**	16	MJ-SC	1q32.2	209501202	*LINC01696*	I	1
**R269**	16	MJ-SC	1q31.1	190713347	*LINC01720*	I	1
**R279**	16	2J-NL	7q32.3	130774908	*LINC-PINT*	I	1
**R153**	16	MJ-SC	3q28	191163068	*LINCR-0002*	I	1
**R367**	45	2J	15q21.3	58732658	*LIPC*	I	1
**R49**	16	MJ-SC	12q13.11	46888980	*OC10028879*	I	1
**R49**	16	MJ-SC	12q13.11	46882890	*OC10028879*	I	1
**R49**	16	MJ-SC	12q13.11	46888985	*OC10028879*	I	1
**R93**	16	2J-COL	10p15.2	3758699	*OC10537636*	I	1
**R93**	16	2J-COL	10p15.2	3760745	*OC10537636*	I	1
**R234**	73	MJ-SC	1q24.1	165503850	*LOC400794*	I	1
**R224**	16	MJ-SC	12p12.1	25203726	*LOC645177*	I	1
**R224**	16	MJ-SC	12p12.1	25203701	*LOC645177*	I	1
**R155**	16	2J-COL	3q28	188301643	*LPP*	I	1
**R155**	16	2J-COL	3q28	188301908	*LPP*	I	1
**R240**	52	MJ-SC	7q36.1	150033174	*LRCC61*	I	1
**R240**	52	MJ-SC	7q36.1	150033154	*LRCC61*	I	1
**R366**	18	2J-COL	2q22.1	141853885	*LRP1B*	I	1
**R366**	18	2J-COL	2q22.1	141683759	*LRP1B*	I	1
**R277**	16	MJ-SC	9q33.3	130222751	*LRSAM1*	I	1
**R33**	16	MJ-SC	8q12.1	56847392	*LYN*	I	1
**R270**	16	MJ-CL	5q14.3	89816616	*LYSMD3*	I	1
**R270**	16	MJ-CL	5q14.3	89812038	*LYSMD3*	E	1
**R66**	16	MJ-SC	20p12.1	15027231	*MACROD2*	I	7
**R66**	16	MJ-SC	20p12.1	14975077	*MACROD2*	I	7
**R66**	16	MJ-SC	20p12.1	14994280	*MACROD2*	I	7
**R66**	16	MJ-SC	20p12.1	14996894	*MACROD2*	I	7
**R66**	16	MJ-SC	20p12.1	15003913	*MACROD2*	I	7
**R66**	16	MJ-SC	20p12.1	14932895	*MACROD2*	I	7
**R66**	16	MJ-SC	20p12.1	14975346	*MACROD2*	I	7
**R66**	16	MJ-SC	20p12.1	15000017	*MACROD2*	I	7

**R66**	16	MJ-SC	20p12.1	15004487	*MACROD2*	I	7
**R66**	16	MJ-SC	20p12.1	14930398	*MACROD2*	I	7
**R66**	16	MJ-SC	20p12.1	15018587	*MACROD2*	I	7
**R71**	16	MJ-SC	20p12.1	15442733	*MACROD2*	I	7
**R71**	16	MJ-SC	20p12.1	15218784	*MACROD2*	I	7
**R72**	16	MJ-SC	20p12.1	15033415	*MACROD2*	I	7
**R72**	16	MJ-SC	20p12.1	15110956	*MACROD2*	I	7
**R77**	33	MJ-SC	20p12.1	15183741	*MACROD2*	I	7
**R77**	33	MJ-SC	20p12.1	15181074	*MACROD2*	I	7
**R152**	52	2J-COL	20p12.1	15236589	*MACROD2*	I	7
**R152**	52	2J-COL	20p12.1	15199051	*MACROD2*	I	7
**R266**	16	MJ-SC	20p12.1	15047101	*MACROD2*	I	7
**R278**	16	MJ-SC	20p12.1	14906751	*MACROD2*	I	7
**R153**	16	MJ-SC	11q21	95841921	*MAML2*	I	1
**R153**	16	MJ-SC	11q21	95804833	*MAML2*	I	1
**R153**	16	MJ-SC	11q21	95818293	*MAML2*	I	1
**R153**	16	MJ-SC	11q21	95867471	*MAML2*	I	1
**R153**	16	MJ-SC	11q21	95770702	*MAML2*	I	1
**R49**	16	MJ-SC	19p13.2	8491725	*MARCH2*	I	1
**R165**	73	MJ-SC	5q12.3	66448504	*MAST4*	E	1
**R221**	16	MJ-SC	2p15	63824153	*MDH1*	I	1
**R69**	16	2J-COL	3q26.2	169040934	*MECOM*	I	1
**R69**	16	2J-COL	3q26.2	169040929	*MECOM*	I	1
**R153**	16	MJ-SC	15q22.31	66190353	*MEGF11*	E	1
**R153**	16	MJ-SC	15q22.31	66202453	*MEGF11*	I	1
**R153**	16	MJ-SC	15q22.31	66236768	*MEGF11*	I	1
**R153**	16	MJ-SC	15q22.31	66223397	*MEGF11*	I	1
**R167**	59	MJ-SC	2p14	66763877	*MEIS1*	I	1
**R217**	16	MJ-SC	Xp22.2	10653310	*MID1*	I	1
**R217**	16	MJ-SC	Xp22.2	10645629	*MID1*	E	1
**R27**	18	2J	14q21.1	38009532	*MIPOL1/TTC6*	I/I	5
**R136**	16	MJ-SC	14q21.1	38013909	*MIPOL1/TTC6*	I/I	5
**R136**	16	MJ-SC	14q21.1	37986383	*MIPOL1/TTC6*	I/I	5
**R241**	16	MJ-SC	14q21.1	38117912	*MIPOL1/TTC6*	I/I	5
**R241**	16	MJ-SC	14q21.1	38076873	*MIPOL1/TTC6*	I/I	5
**R241**	16	MJ-SC	14q21.1	38117769	*MIPOL1/TTC6*	I/I	5
**R241**	16	MJ-SC	14q21.1	38124598	*MIPOL1/TTC6*	I/I	5
**R241**	16	MJ-SC	14q21.1	38179398	*MIPOL1/TTC6*	I/I	5
**R241**	16	MJ-SC	14q21.1	38109161	*MIPOL1/TTC6*	I/I	5
**R241**	16	MJ-SC	14q21.1	38122149	*MIPOL1/TTC6*	I/I	5
**R269**	16	MJ-SC	14q21.1	37900956	*MIPOL1/TTC6*	I/I	5
**R269**	16	MJ-SC	14q21.1	37992826	*MIPOL1/TTC6*	I/I	5
**R269**	16	MJ-SC	14q21.1	38178009	*MIPOL1/TTC6*	I/I	5
**R377**	16	MJ-SC	14q21.1	37886925	*MIPOL1/TTC6*	I/I	5
**R377**	16	MJ-SC	14q21.1	37918844	*MIPOL1/TTC6*	I/I	5
**R377**	16	MJ-SC	14q21.1	38011105	*MIPOL1/TTC6*	I/I	5
**R266**	16	MJ-SC	1q32.2	209603003	*MIR205HG*	I	1
**R266**	16	MJ-SC	1q32.2	209603022	*MIR205HG*	I	1
**R266**	16	MJ-SC	1q32.2	209605369	*MIR205HG*	I	1
**R266**	16	MJ-SC	1q32.2	209604176	*MIR205HG*	I	1

**R269**	16	MJ-SC	7q32.3	131159936	*MKLN1*	I	1
**R209**	45	2J-COL	2q23.2	150427072	*MMADHC*	I	1
**R209**	45	2J-COL	2q23.2	150427100	*MMADHC*	I	1
**R341**	16	2J-NL	11q22.2	102737143	*MMP12*	E	1
**R133**	16	MJ-SC	8q24.3	142479037	*MROH5*	I	1
**R39**	16	MJ-CL	9q31.1	103229773	*ANTD3-TMEI*	I	1
**R168**	45	MJ-CL	4q13.3	74982515	*MTHFD2L*	I	1
**R216**	16	MJ-SC	16p13.11	15833551	*MYH11*	I	1
**R216**	16	MJ-SC	16p13.11	15833546	*MYH11*	I	1
**R216**	16	MJ-SC	16p13.11	15850319	*MYH11*	E	1
**R204**	56	MJ-SC	13q33.3	109610852	*MYO16*	I	1
**R204**	56	MJ-SC	13q33.3	109617202	*MYO16*	E	1
**R204**	56	MJ-SC	13q33.3	109613367	*MYO16*	I	1
**R204**	56	MJ-SC	13q33.3	109609921	*MYO16*	I	1
**R204**	56	MJ-SC	13q33.3	109671789	*MYO16*	I	1
**R204**	56	MJ-SC	13q33.3	109564847	*MYO16*	I	1
**R204**	56	MJ-SC	13q33.3	109587393	*MYO16*	I	1
**R204**	56	MJ-SC	13q33.3	109573556	*MYO16*	I	1
**R204**	56	MJ-SC	13q33.3	109599665	*MYO16*	I	1
**R204**	56	MJ-SC	13q33.3	109582799	*MYO16*	I	1
**R204**	56	MJ-SC	13q33.3	109576992	*MYO16*	I	1
**R344**	45	2J-NL	1q24.3	171606066	*MYOC*	I	1
**R66**	16	MJ-SC	13q26.31	174790714	*NAADLADL2*	I	2
**R66**	16	MJ-SC	13q26.31	174916573	*NAADLADL2*	I	2
**R66**	16	MJ-SC	13q26.31	174778159	*NAADLADL2*	I	2
**R173**	18	2J-NL	3q26.31	175461851	*NAADLADL2*	I	2
**R173**	18	2J-NL	3q26.31	175514309	*NAADLADL2*	I	2
**R66**	16	MJ-SC	3q26.31	174910984	*NAADLADL3*	I	1
**R66**	16	MJ-SC	13q26.31	174916905	*NAADLADL3*	I	1
**R66**	16	MJ-SC	13q26.31	174911625	*NAADLADL3*	I	1
**R153**	16	MJ-SC	3q26.31	175136454	*NAALADL2*	I	1
**R41**	16	MJ-SC	2q33.2	203882995	*NBEAL1*	I	1
**R236**	16	MJ-SC	8q22.3	102951451	*NCALD*	I	1
**R236**	16	MJ-SC	8q22.3	103003034	*NCALD*	I	1
**R164**	16	MJ-SC	5q35.1	172090257	*NEURL1B*	I	1
**R267**	16	2J-NL	16p11.2	28964750	*NFATC2IP*	I	1
**R283**	16	MJ-SC	2q31.2	178198214	*NFE2L2*	I	1
**R283**	16	MJ-SC	2q31.2	178189233	*NFE2L2*	I	1
**R28**	16	MJ-CL	19p13.3	3387974	*NFIC*	I	1
**R28**	16	MJ-CL	19p13.3	3425834	*NFIC*	I	1
**R28**	16	MJ-CL	19p13.3	3384777	*NFIC*	I	1
**R28**	16	MJ-CL	19p13.3	3441402	*NFIC*	I	1
**R28**	16	MJ-CL	19p13.3	3433667	*NFIC*	E	1
**R28**	16	MJ-CL	19p13.3	3431302	*NFIC*	I	1
**R52**	16	MJ-SC	11q24.3	129755159	*NFRKB*	I	1
**R52**	16	MJ-SC	11q24.3	129755188	*NFRKB*	I	1
**R263**	18	MJ-SC	3q26.31	173355918	*NLGN1*	I	1
**R323**	39	2J-COL	1q24.2	169181322	*NME7*	I	1
**R323**	39	2J-COL	1q24.2	169181306	*NME7*	I	1
**R277**	16	MJ-SC	17q24.2	65733128	*NOL11*	I	1

**R136**	16	MJ-SC	4q31.23	149102742	*NR3C2*	I	1
**R278**	16	MJ-SC	9q33.3	127260979	*NR5A1*	I	1
**R278**	16	MJ-SC	9q33.3	127260952	*NR5A1*	I	1
**R333**	18	MJ-CL	8p12	32470812	*NRG1*	I	1
**R333**	18	MJ-CL	8p12	32495266	*NRG1*	I	1
**R333**	18	MJ-CL	8p12	32472972	*NRG1*	I	1
**R333**	18	MJ-CL	8p12	32428884	*NRG1*	I	1
**R333**	18	MJ-CL	8p12	32393443	*NRG1*	I	1
**R333**	18	MJ-CL	8p12	32394819	*NRG1*	I	1
**R206**	16	MJ-SC	5q31.2	139334420	*NRG2*	I	1
**R206**	16	MJ-SC	5q31.2	139235043	*NRG2*	I	1
**R65**	18	2J-COL	21q11.2	16367684	*NRIP1*	I	1
**R65**	18	2J-COL	21q11.2	16367672	*NRIP1*	I	1
**R222**	16	MJ-CL	4p16.3	1956512	*NSD2*	I	1
**R348**	16	MJ-SC	8q24.13	126206376	*NSMCE2*	I	1
**R348**	16	MJ-SC	8q24.13	126258239	*NSMCE2*	I	1
**R153**	16	MJ-SC	17q11.2	28459938	*NSRP1*	I	1
**R153**	16	MJ-SC	17q11.2	28488771	*NSRP1*	I	1
**R153**	16	MJ-SC	17q11.2	28459911	*NSRP1*	I	1
**R33**	16	MJ-SC	1q32.1	205277102	*NUAK2*	I	1
**R89**	16	MJ-SC	8p21.1	27929195	*NUGGC*	I	1
**R225**	16	2J-NL	1p32.3	52865497	*ORC1*	I	1
**R33**	16	MJ-SC	10q11.23	51027231	*PARG*	E	1
**R325**	16	MJ-SC	Xq21.32	91841433	*PCDH11X*	I	1
**R379**	16	MJ-CL	9q21.13	78601101	*PCSK5*	E	1
**R379**	16	MJ-CL	9q21.13	78598484	*PCSK5*	I	1
**R238**	18	MJ-SC	1p31.3	66338203	*PDE4B*	I	1
**R89**	16	MJ-SC	5q12.1	59116045	*PDE4D*	I	1
**R224**	16	MJ-SC	11q22.3	104024395	*PDGFD*	I	1
**R224**	16	MJ-SC	11q22.3	103964393	*PDGFD*	I	1
**R268**	16	MJ-CL	1p36.21	13912938	*PDPN*	I	1
**R268**	16	MJ-CL	1p36.21	13932764	*PDPN*	I	1
**R268**	16	MJ-CL	1p36.21	13932806	*PDPN*	I	1
**R49**	16	MJ-SC	10p15.1	6260071	*PFKFB3*	I	1
**R49**	16	MJ-SC	10p15.1	6236802	*PFKFB3*	I	1
**R67**	16	MJ-SC	17q12	37837140	*PGAP3*	I	1
**R238**	18	MJ-SC	20q11.23	34532817	*PHF20*	I	1
**R238**	18	MJ-SC	20q11.23	34514952	*PHF20*	I	1
**R166**	16	MJ-SC	13q22.1	73549498	*PIBF1*	I	1
**R19**	31	MJ-SC	18q21.33	59852244	*PIGN*	I	1
**R19**	31	MJ-SC	18q21.33	59852221	*IGN/KIAA146*	I/E	1
**R184**	16	MJ-SC	2q33.1	198727867	*PLCL1*	I	1
**R184**	16	MJ-SC	2q33.1	198687720	*PLCL1*	I	1
**R33**	16	MJ-SC	9p24.1	5442127	*PLGRKT*	I	1
**R33**	16	MJ-SC	9p24.1	5441945	*PLGRKT*	I	1
**R33**	16	MJ-SC	9p24.1	5418033	*PLGRKT*	I	1
**R153**	16	MJ-SC	3q24	146308844	*PLSCR5*	I	1
**R153**	16	MJ-SC	3q24	146308845	*PLSCR5*	I	1
**R270**	16	MJ-CL	5q14.3	89793094	*POLR3G*	I	1
**R216**	16	MJ-SC	1p36.21	13161978	*PRAMEF34P*	E	1

**R228**	73	2J-NL	14q23.1	61967842	*PRKCH*	I	1
**R228**	73	2J-NL	14q23.1	62012978	*PRKCH*	I	1
**R262**	18	2J	10p14	11932852	*PROSER2-AS1*	I	1
**R344**	45	2J-NL	1q24.3	171560468	*PRRC2C*	I	1
**R48**	16	MJ-SC	10q23.31	89692955	*PTEN*	E	1
**R48**	16	MJ-SC	10q23.31	89692948	*PTEN*	E	1
**R253**	16	MJ-SC	4q21.3	87592400	*PTPN13*	I	1
**R48**	16	MJ-SC	9p23	9795312	*PTPRD*	I	1
**R48**	16	MJ-SC	9p23	9795310	*PTPRD*	I	1
**R133**	16	MJ-SC	8q24.21	128951223	*PVT1*	I	2
**R133**	16	MJ-SC	8q24.21	128973206	*PVT1*	I	2
**R133**	16	MJ-SC	8q24.21	128956914	*PVT1*	I	2
**R133**	16	MJ-SC	8q24.21	128952469	*PVT1*	I	2
**R133**	16	MJ-SC	8q24.21	128912182	*PVT1*	I	2
**R133**	16	MJ-SC	8q24.21	128955253	*PVT1*	I	2
**R133**	16	MJ-SC	8q24.21	128958407	*PVT1*	I	2
**R187**	16	MJ-SC	8q24.21	129020069	*PVT1*	I	2
**R21**	18	2J-COL	15q22.31	66166367	*RAB11A*	I	1
**R21**	18	2J-COL	15q22.31	66166358	*RAB11A*	I	1
**R41**	16	MJ-SC	4q31.1	140381150	*RAB33B*	I	1
**R31**	16	MJ-SC	14q24.1	68932393	*RAD51B*	I	1
**R38**	33	MJ-SC	14q24.1	68674773	*RAD51B*	I	1
**R277**	16	MJ-SC	16p13.3	6599606	*RBFOX1*	I	1
**R277**	16	MJ-SC	16p13.3	6632761	*RBFOX1*	I	1
**R275**	31	MJ-CL	8q22.3	105130645	*RIMS2*	I	1
**R275**	31	MJ-CL	8q22.3	105028616	*RIMS2*	I	1
**R275**	31	MJ-CL	8q22.3	105142897	*RIMS2*	I	1
**R275**	31	MJ-CL	8q22.3	105106250	*RIMS2*	I	1
**R275**	31	MJ-CL	8q22.3	105142646	*RIMS2*	I	1
**R275**	31	MJ-CL	8q22.3	105123452	*RIMS2*	I	1
**R275**	31	MJ-CL	8q22.3	105032248	*RIMS2*	I	1
**R275**	31	MJ-CL	8q22.3	105140287	*RIMS2*	I	1
**R275**	31	MJ-CL	8q22.3	105133280	*RIMS2*	I	1
**R275**	31	MJ-CL	8q22.3	105054693	*RIMS2*	I	1
**R275**	31	MJ-CL	8q22.3	104542763	*RIMS2*	I	1
**R275**	31	MJ-CL	8q22.3	105136066	*RIMS2*	I	1
**R275**	31	MJ-CL	8q22.3	105139843	*RIMS2*	I	1
**R89**	16	MJ-SC	9q21.32	86605805	*RMI1*	I	1
**R145**	33	MJ-CL	19q13.33	49994866	*RPL13A*	I	1
**R145**	33	MJ-CL	19q13.33	49994844	*RPL13A*	I	1
**R55**	16	MJ-SC	3q25.32	157918340	*RSRC1*	I	1
**R179**	16	MJ-CL	8q24.12	119633333	*SAMD12*	I	1
**R179**	16	MJ-CL	8q24.12	119489095	*SAMD12*	I	1
**R179**	16	MJ-CL	8q24.12	119488323	*SAMD12*	I	1
**R179**	16	MJ-CL	8q24.12	119487686	*SAMD12*	I	1
**R377**	16	MJ-SC	11p15.4	9982040	*SBF2*	I	1
**R377**	16	MJ-SC	11p15.4	9983752	*SBF2*	I	1
**R377**	16	MJ-SC	11p15.4	9959285	*SBF2*	I	1
**R377**	16	MJ-SC	11p15.4	9900221	*SBF2*	I	1
**R377**	16	MJ-SC	11p15.4	9817784	*SBF2*	I	1

**R88**	16	2J-NL	8p21.1	27506733	*SCARA3*	I	1
**R215**	16	MJ-CL	5q33.3	155728712	*SGCD*	I	1
**R144**	16	MJ-SC	8p22	14601023	*SGCZ*	I	1
**R144**	16	MJ-SC	8p22	14601008	*SGCZ*	I	1
**R144**	16	MJ-SC	8p22	14606679	*SGCZ*	I	1
**R41**	16	MJ-SC	6q23.2	134518164	*SGK1*	I	2
**R264**	33	2J-NL	6q23.2	134517053	*SGK1*	I	2
**R264**	33	2J-NL	6q23.2	134608057	*SGK1*	I	2
**R135**	16	2J-COL	2q37.2	235860641	*SH3BP4*	E	1
**R221**	16	MJ-SC	19p13.3	4382144	*SH3GL1*	I	1
**R248**	18	2J-NL	11q23.1	111551561	*SIK2*	I	1
**R248**	18	2J-NL	11q23.1	111551522	*SIK2*	I	1
**R220**	16	MJ-CL	4q21.3	87769849	*SLC10A6*	I	1
**R192**	18	MJ-SC	15q21.2	50501894	*SLC27A2*	I	1
**R192**	18	MJ-SC	15q21.2	50501871	*SLC27A2*	I	1
**R324**	16	MJ-SC	5p15.33	1238482	*SLC6A18*	I	1
**R324**	16	MJ-SC	5p15.33	1224437	*SLC6A19*	I	1
**R218**	16	MJ-SC	5p15.33	1207113	*SLC6A3*	I	1
**R269**	16	MJ-SC	1p34.1	44490954	*SLC6A9*	I	1
**R342**	82	MJ-CL	16q22.1	67304018	*SLC9A5*	I	1
**R89**	16	MJ-SC	5q35.1	168641779	*SLIT3*	I	1
**R92**	31	2J-COL	15q22.33	67431401	*SMAD3*	I	1
**R92**	31	2J-COL	15q22.33	67431432	*SMAD3*	I	1
**R255**	18	2J-NL	3q29	196234932	*SMCO1*	E	1
**R255**	18	2J-NL	3q29	196239676	*SMCO1*	I	1
**R58**	18	MJ-CL	1q32.3	214466362	*SMYD2*	I	1
**R57**	70	2J-COL	1q44	246018370	*SMYD3*	I	2
**R153**	16	MJ-SC	1q44	246202718	*SMYD3*	I	2
**R57**	70	2J-COL	1q44	245926204	*SMYD4*	I	1
**R138**	16	2J-COL	19q13.2	41270213	*SNRPA*	I	1
**R138**	16	2J-COL	19q13.2	41270251	*SNRPA*	I	1
**R388**	16	MJ-SC	20q11.21	32026439	*SNTA1*	I	1
**R342**	82	MJ-CL	16p13.3	12305801	*SNX29*	I	1
**R86**	16	MJ-SC	5q33.1	151046934	*SPARC*	I	1
**R86**	16	MJ-SC	5q33.1	151046949	*SPARC*	I	1
**R39**	16	MJ-CL	9p24.1	4639325	*SPATA6L*	I	1
**R263**	18	MJ-SC	8q11.21	48553952	*SPIDR*	I	1
**R263**	18	MJ-SC	8q11.21	48514754	*SPIDR*	I	1
**R46**	45	2J-NL	5q32	147469596	*SPINK5*	I	1
**R78**	16	2J	17q22	56078358	*SRFS1*	E	1
**R163**	31	2J-NL	12p12.1	26386424	*SSPN*	E	1
**R161**	59	MJ-SC	6p24.3	7284873	*SSR1*	E	1
**R59**	33	2J	3p22.3	36498343	*STAC*	I	1
**R221**	16	MJ-SC	19p13.3	4336052	*STAP2*	I	1
**R83**	33	MJ-SC	7q21.13	89835503	*STEAP2*	I	1
**R216**	16	MJ-SC	2q24.3	169095396	*STK39*	I	1
**R221**	16	MJ-SC	Xp22.31	7247855	*STS*	I	1
**R276**	16	2J-COL	22q13.1	39132336	*SUN2*	E	1
**R276**	16	2J-COL	22q13.1	39132316	*SUN2*	E	1
**R164**	16	MJ-SC	9q31.3	114869927	*SUSD1*	I	1

**R222**	16	MJ-CL	4p16.3	1736508	*TACC3*	I	1
**R269**	16	MJ-SC	2q24.2	159937816	*TANC1*	I	1
**R230**	16	MJ-SC	22q12.2	30707071	*TBC1D10A*	I	1
**R224**	16	MJ-SC	10q26.3	132897205	*TCERG1L*	I	1
**R165**	73	MJ-SC	4q13.1	65150940	*TECRL*	I	1
**R59**	39	MJ-CL	5q34	167178055	*TENM2*	I	1
**R324**	16	MJ-SC	5p15.33	1279521	*TERT*	E	1
**R324**	16	MJ-SC	5p15.33	1280549	*TERT*	I	1
**R324**	16	MJ-SC	5p15.33	1256079	*TERT*	I	1
**R324**	16	MJ-SC	5p15.33	1256828	*TERT*	I	1
**R324**	16	MJ-SC	5p15.33	1258146	*TERT*	I	1
**R91**	31	MJ-CL	6p21.1	41679641	*TFEB*	I	1
**R91**	31	MJ-CL	6p21.1	41679669	*TFEB*	I	1
**R91**	31	MJ-CL	6p21.1	41653954	*TFEB*	E	1
**R176**	18	2J-NL	2q12.1	105893192	*TGFBRAP1*	I	1
**R221**	16	MJ-SC	6p21.1	44112268	*TMEM63B*	I	1
**R221**	16	MJ-SC	6p21.1	44111037	*TMEM63B*	I	1
**R351**	45	2J-COL	9q33.1	117871667	*TNC*	I	1
**R351**	45	2J-COL	9q33.1	117871675	*TNC*	I	1
**R288**	16	MJ-SC	8p21.3	22922861	*TNFRSF110B*	I	1
**R288**	16	MJ-SC	8p21.3	22896097	*TNFRSF110B*	I	1
**R174**	59	2J-NL	3q28	189589485	*TP63*	I	5
**R231**	45	2J-NL	3q28	189585530	*TP63*	I	5
**R252**	73	2J-NL	3q28	189595951	*TP63*	I	5
**R338**	16	2J	3q28	189498697	*TP63*	I	5
**R373**	58	2J	3q28	189594960	*TP63*	I	5
**R141**	16	MJ-SC	16p13.3	3737872	*TRAP1*	I	1
**R79**	16	MJ-SC	20q13.3	51962925	*TSHZ2*	I	2
**R139**	16	2J	20q13.2	51699755	*TSHZ2*	I	2
**R266**	16	MJ-SC	17q23.1	57951828	*TUBD1*	I	1
**R269**	16	MJ-SC	13q34	113189775	*TUBGCP3*	I	1
**R269**	16	MJ-SC	13q34	113200037	*TUBGCP3*	E	1
**R153**	16	MJ-SC	6p21.31	35475484	*TULP1*	I	1
**R41**	16	MJ-SC	Xq24	118710489	*UBE2A*	I	1
**R164**	16	MJ-SC	9q31.3	114695557	*UGCG*	E	1
**R85**	16	MJ-SC	1p32.3	55641834	*USP24*	I	1
**R85**	16	MJ-SC	2q35	219369570	*USP37*	I	1
**R223**	45	2J-NL	2q37.1	234416054	*USP40*	I	1
**R131**	18	MJ-CL	17q23.1	57852019	*VMP1*	I	2
**R131**	18	MJ-CL	17q23.1	57895852	*VMP1*	I	2
**R274**	45	2J-NL	17q23.1	57823844	*VMP1*	I	2
**R274**	45	2J-NL	17q23.1	57867967	*VMP1*	I	2
**R221**	16	MJ-SC	8q22.2	100192304	*VPS13B*	I	1
**R221**	16	MJ-SC	8q22.2	100205405	*VPS13B*	I	1
**R20**	18	2J-NL	14q32.31	102632969	*WDR20*	I	1
**R20**	18	2J-NL	14q32.31	102646247	*WDR20*	I	1
**R72**	16	MJ-SC	3p14.3	55502498	*WNT5A*	E	1
**R67**	16	MJ-SC	16q23.1	79145226	*WWOX*	I	1
**R67**	16	MJ-SC	16q23.1	79006948	*WWOX*	I	1
**R254**	73	MJ-SC	3q13.31	114194684	*ZBTB20*	I	1

**R254**	73	MJ-SC	3q13.31	114181688	*ZBTB20*	I	1
**R41**	16	MJ-SC	18q21.1	45607692	*ZBTB7C*	I	1
**R41**	16	MJ-SC	18q21.1	45607712	*ZBTB7C*	I	1
**R41**	16	MJ-SC	18q21.1	45580444	*ZBTB7C*	I	1
**R225**	16	2J-NL	1p32.3	52896975	*ZCCHC*	E	1
**R224**	16	MJ-SC	6p21.2	38093525	*ZFAND3*	I	1
**R243**	73	2J-COL	10q22.3	81028224	*ZMIZ1*	I	1
**R243**	73	2J-COL	10q22.3	81028238	*ZMIZ1*	I	1
**R41**	16	MJ-SC	18q21.32	56551967	*ZNF532*	I	1
**R269**	16	MJ-SC	19q13.43	56978888	*ZNF667*	I	1
**R269**	16	MJ-SC	19q13.43	56979230	*ZNF667*	I	1
**R71**	16	MJ-SC	19q13.43	58813491	*ZNF8*	E	1
**R144**	16	MJ-SC	19p13.2	12187685	*ZNF844*	I	1
**R71**	16	MJ-SC	19q13.43	58801482	*ZNF9*	I	1

I=intron

E=exon

List of human genes with HPV integrations in sereval cases of cervical cancers

**Genes with HPV integration in**		
**2 cases n=16**	**5 cases n=2**	**7 cases n=1**
AKR1C3	*MIPOL1/TTC6*	*MACROD2*
ATRNL1	*TP63*	
BCL11B		
CEACAM5		
CEACAM6		
CREBBP		
DIAPH2		
ERBB2		
FHIT		
LEPREL1		
NAADLADL2		
PVT1		
SGK1		
SMYD3		
TSHZ2		
VMP1		

nf-VIF: A Nextflow-based Virus Insertion Finder

Background

Human papillomavirus (HPV) infection remains the first cause of cervical cancer, one of the most common cancers in women worldwide. HPV infection is also involved in numerous and various types of cancer in penis, vagina, anus, and head and neck. In clinics, a persistent infection with HPV high-risk genotypes is associated with cancer progression and response to treatment. Therefore, being able to genotype HPVs in cancer samples, and to characterize the insertion sites and the relying mechanisms is of high interest in clinical research and diagnosis. Thanks to the emergence of next-generation sequencing techniques, new approaches were developed to answer these questions. As any assay based on sequencing technology, these approaches requires appropriate bioinformatics methods and tools to check the quality of the samples, and to process and analyse the sequencing data.

In addition, the portability, scalability and reproducibility of bioinformatics pipelines is a well-known problem within the scientific community. Most of the time, bioinformatic analysis pipelines are designed for a single project execution, and therefore often require local customisation or modification of hard-coded part of the sources, which inevitably lead to reproducibility issues. The development of FAIR (Findable, Accessible, Interoperable and Reusable) methods and tools is therefore a priority and a need to address these issues.

Here, we present a new bioinformatics pipeline for virus insertion detection which is based on our previous experience and methodology on NGS-based HPV genotyping [1]. This pipeline relies on a dedicated workflow which briefly i) check the sample quality ii) detect the virus genotypes and ii) infer the precise insertion loci using a local mapping strategy. The pipeline is built following good coding practices and the Nextflow workflow management system, thus ensuring a high portability, reproducibility and scalability of the workflow.

Workflow description

Sequencing reads are first trimmed with the *TrimGalore* software to remove any adapter sequences at the 3' end of the reads, as well as low quality bases (--quality 20) and sort sequences (--lengths 20). While this step is optional, it is highly recommended to avoid noise in the downstream analysis and in the detection of soft-clipped reads.

FastQC is then used to assess the overall sequencing quality of trimmed sequences. It provides information about the quality score distribution across the reads, the per base sequence content (%T/A/G/C), any remaining adapter contamination and other overrepresented sequences.

Once the data are clean, the *Bowtie2* software [2] is then used to :

1) Align all the trimmed reads from end-to-end against three control genes (*KLK3*, *GAPDH*, *RAB7A*) in order to estimate the virus load/burden.
2) Align all the trimmed reads from end-to-end against a list of virus references genomes.
3) Aligned all the trimmed reads using a local mapping algorithm against the, at most 3, major virus strains previously detected, in order to precisely define the virus insertion sites.

Once the data are aligned, we then use an in-house Python script to extract :

1) the breakpoint positions in the virus using 5’/3’ soft-clipped reads.
2) the corresponding human sequences in these human/virus chimeric reads

Finally, the *Blat* software is used to have a high accuracy alignment (>=90% similarity of length >=25 bases) of these extracted human sequences to extract precise insertion sites positions on the human genome. From these results, we then prioritize the detected breakpoints based on i) the mappability at this locus, ii) the number of supporting reads iii) the presence of both 5’/3’ breakpoints on the same chromosome associated with the same virus strain.

All results are filtered and gathered in a user-friendly report through the MultiQC software [3]. The report can be done for all the samples together or for each individual sample. Briefly, it proposes a synthetic view of the data quality, alignment results, and detected virus genotypes. For each genotype, the reads coverage and the position of each detected breakpoint with the number of supporting reads is represented through dynamic view of the viral genome and its gene annotations.

Implementation

This pipeline is based on the Nextflow management system [4]. As other workflow management systems, Nextflow distinguishes the computing infrastructure requirement to the analysis workflow itself. It therefore natively introduces a high degree of portability and reproducibility of the analysis. As an example, most High Performance Computing schedulers are natively supported by Nextflow such as SLURM, LSF, PBS, Torque, etc. It also has built-in support for deployment using container technologies such as Conda, Docker, and Singularity. In addition, the baseline of the pipeline was written following best-coding practices promoted by the *nf-core* community. The *nf-core* is a community of almost 90 developers representing almost 30 organisations that provides state-of-the-art bioinformatics analysis pipelines and proposed strict guidelines for their development and the usage of the Nextflow management system.

The nf-VIF pipeline therefore inherits from these functionalities and provides support for Conda or Singularity usage. In practice, it means that the end-users do not need to manage the pipeline dependencies which are already provided in containers and/or recipes to create them. It can be run on a local infrastructure such a laptop, or on a computational cluster to process all samples in parallel. Each step of the pipeline can be managed to define the required resources in terms of CPUs, memory (RAM) and time. All these points are key features of reproducible research, and are especially important in the context of tools dedicated to the analysis of clinical and/or diagnosis data. Thus, nf-VIF provides a high level of reproducibility and tracability.

Usage example

The pipeline comes with a complete manual available online describing all pipeline options and outputs, as well as a test dataset to ensure that the pipeline is properly set up. By default, nf-VIF can be used in a very straightforward way by simply specifying a path to input sequencing data or a text file listing all samples to analyse (sample plan).
Note that there is no restriction on the number of samples to analyse. All analysis will be managed with a queue system according to the resources available on the infrastructure.

```
nextflow run main.nf --reads ‘*_R{1,2}.fastq.gz’ -profile cluster,conda
```

Note that specifying the options `-profile cluster,conda` will automatically build a conda environment and run the analysis on the cluster architecture. For more details about available profile, see the manual of the pipeline.

Importantly, nf-VIF is not restricted to any sequencing kits or capture protocol. All options, control regions and virus references can be updated by the end-users in command line. Here is a simple exemple to customize the list of reference virus and of control sequences.

```
nextflow run main.nf --reads ‘*_R{1,2}.fastq.gz’ -profile cluster,conda --fasta_hpv ‘myHPVref.fa’ --fasta_ctrl ‘myCtrl.fa’
```

Availability

The pipeline is freely available at https://github.com/bioinfo-pf-curie/nf-vif/ under the CECILL licence. More information are available on the website.

Acknowledgement

We would like to thank our colleagues from Hôpital Européen Georges Pompidou (HEGP), and especially M. Wack, B. Rance, H. Péré and D. Veyer for useful discussion about HPV insertion analysis.

References

[1] Holmes, A., Lameiras, S., Jeannot, E. *et al.* Mechanistic signatures of HPV insertions in cervical carcinomas. *npj Genomic Med* 1, 16004 (2016) doi:10.1038/npjgenmed.2016.4
[2] Langmead B., Salzberg S. Fast gapped-read alignment with Bowtie 2. Nature Methods, 9:357-359 (2012)
[3] Ewels P., Magnusson M., Lundin S., Käller M. MultiQC: summarize analysis results for multiple tools and samples in a single report, *Bioinformatics*. 32-19:3047–3048 (2016) doi:10.1093/bioinformatics/btw354
[4] Di Tommaso, P., Chatzou, M., Floden, E. *et al.* Nextflow enables reproducible computational workflows. *Nat Biotechnol* 35, 316–319 (2017) doi:10.1038/nbt.3820
[5] Ewels P., Peltzer A., Fillinger S., Alneberg J., Patel H., Wilm A., Garcia MU., Di Tommaso P., Nahnsen S. nf-core: Community curated bioinformatics pipelines. bioRxiv. doi.org/10.1101/610741
